# Evaluating the Efficacy and Safety of TACE Combined with Iodine-125 Brachytherapy Versus TACE Monotherapy for Hepatocellular Carcinoma: A Systematic Review and Meta-Analysis

**DOI:** 10.3390/jcm15062267

**Published:** 2026-03-17

**Authors:** Israa Alhashimi, Abeer Abdullah Hamid, Dana Elkhalifa, Sohaib Zoghoul, Ali Barah, Mohamed Izham Mohamed Ibrahim

**Affiliations:** 1 Clinical Imaging Department, Hamad Medical Corporation, Doha P.O. Box 3488, Qatarszoghoul@hamad.qa (S.Z.); abarah@hamad.qa (A.B.); 2 College of Pharmacy, University of AI Maarif, Al Anbar 31001, Iraq; abeer.abdullah@uoa.edu.iq; 3 Department of Pharmacy, Aspetar Orthopedics and Sports Medicine Hospital, Doha P.O. Box 29222, Qatar; 4 College of Health and Life Sciences, Hamad bin Khalifa University, Doha P.O. Box 34110, Qatar; 5 College of Pharmacy, QU Health, Qatar University, Doha P.O. Box 2713, Qatar

**Keywords:** hepatocellular carcinoma, TACE, iodine-125, brachytherapy, survival outcomes, hepatitis B virus

## Abstract

**Background/Objectives:** This review and meta-analysis assessed whether combining transarterial chemoembolization (TACE) with iodine-125 brachytherapy (I-125 brachytherapy) offers greater efficacy and safety than TACE alone in treating hepatocellular carcinoma (HCC). **Methods:** PubMed, EMBASE, the Cochrane Library, Scopus, and Web of Science were searched for articles published between 1 January 2010 and 30 November 2023. Eligible studies compared TACE with and without I-125 brachytherapy from randomized controlled trials (RCTs) and non-randomized comparative studies published in English. The primary outcome was overall survival (OS) at 1, 2, and 3 years. The secondary outcomes included progression-free survival (PFS), overall response rate (ORR), disease control rate (DCR), and adverse events. ROB-2 and ROBINS-I tools were used to assess study quality. **Results:** Eighteen studies (*n* = 1872 patients) were included. All 18 studies originated from China, with the majority being retrospective cohorts (*n* = 16), one non-randomized prospective study, and one RCT. Compared with TACE alone, TACE + I-125 brachytherapy significantly improved OS at 1 year (OR = 3.64, 95% CI: 2.92–4.55), 2 years (OR = 3.93, 95% CI: 2.29–6.77), and 3 years (OR = 4.12, 95% CI: 2.24–7.56). The tumor response rates, including the ORR and DCR, were also significantly higher in the combination group. Subgroup analysis revealed that the survival benefit was maintained in studies without systemic chemotherapy (OR = 3.68, 95% CI: 2.89–4.70) and in studies with systemic chemotherapy (OR = 4.13, 95% CI: 1.69–10.09). Although larger effect estimates were observed with low-dose I-125 brachytherapy (<80 Gy; OR = 8.55, 95% CI: 4.32–16.92) compared to high-dose (≥100 Gy; OR = 2.87, 95% CI: 2.05–4.00), this finding is hypothesis-generating rather than conclusive and should be interpreted cautiously as it is based on only three studies. Adverse event rates were comparable between groups. GRADE assessment indicated low to very low certainty for all outcomes, primarily due to the retrospective nature of most included studies. **Conclusions:** TACE combined with I-125 brachytherapy was associated with improved survival and tumor response without a statistically significant increase in adverse events. High-quality, multicenter RCTs are warranted to confirm these results.

## 1. Introduction

Hepatocellular carcinoma (HCC) is the most common primary liver malignancy (75–85% of cases), arising from hepatocytes, the liver’s primary functional units [1,2]. Development is most often observed in individuals with underlying chronic liver disorders, including cirrhosis, viral hepatitis (B or C), alcoholic liver disease, or non-alcoholic fatty liver disease [1,3,4]. As the third leading cause of tumor-associated mortality globally, HCC represents a serious public health challenge [2,5,6]. This burden is considerable for low- and middle-income countries, though higher-income countries, including those in the Gulf Region, are also experiencing this rising burden [4].

Optimizing HCC therapeutic strategies is highly important in modern times. A diverse array of multidisciplinary approaches have been developed and are continuously being refined to address the HCC burden [7,8,9,10,11]. Curative therapies, such as liver transplantation and surgical resection, are beneficial in up to 30–40% of patients, with the majority of patients relying on locoregional therapies and other supportive interventions [8]. Among these, TACE has emerged as a cornerstone therapeutic modality. TACE facilitates targeted delivery of chemotherapeutic agents directly to the tumor site while obstructing the arterial blood supply to the tumor vasculature [12,13]. For appropriately selected patients with intermediate-stage HCC (BCLC stage B)—typically with preserved liver function and without vascular invasion or extrahepatic spread—TACE is a standard first-line liver-directed therapy intended to achieve tumor control and prolong overall survival [12]. In addition, effective TACE implementation is beneficial for managing advanced stages by controlling symptoms and prolonging overall survival. It also serves as a bridging therapy for early and intermediate stages, increasing a patient’s eligibility for curative therapies and expanding HCC treatment options [7,12,13].

Portal Vein Tumor Thrombosis (PVTT) is a clinically important complication of HCC and is generally associated with poor prognosis, reflecting aggressive tumor biology, impaired portal flow, and limited feasibility of locoregional control [14,15,16,17]. It affects a significant proportion of patients and often leads to worse outcomes [18,19,20]. PVTT often limits curative treatment options; liver transplantation is usually not feasible, and surgical resection remains controversial and highly selection-dependent [14,15,16,17]. Other treatments, such as ablation and chemoembolization, are also less effective; however, newer approaches, including the addition of I-125 brachytherapy, show promise for treating HCC with or without PVTT [21].

Given the limitations of monotherapies, interest in exploring optimal combination therapies to enhance treatment efficacy and safety outcomes in HCC patients has increased [10,11,12,22,23]. One such combination under scrutiny is the integration of brachytherapy (e.g., I-125 brachytherapy) with TACE [24,25,26,27]. For example, I-125 brachytherapy is a minimally invasive treatment that delivers targeted doses of radioactive I-125 brachytherapy to the tumor site. While I-125 brachytherapy can be used as a primary monotherapy, the combined approach aims to augment the local therapeutic effects of TACE and ultimately has the potential to improve tumor response rates, delay disease progression, and increase overall survival [24,25].

Managing HCC has become increasingly complex with advances in treatment modalities, particularly ongoing updates to TACE protocols and diverse combinations of adjuvant treatments and antitumor agents used in studies with varying methodological quality [13,24]. Previous systematic reviews have examined survival and tumor control outcomes in HCC patients subjected to TACE plus I-125 brachytherapy or TACE alone [24,25]. However, these reviews are no longer current, and an updated systematic review is warranted to synthesize emerging evidence on advancements in treatment modalities and provide a comprehensive outcome analysis. Such an extensive analysis has not yet been performed. In particular, the latest review addressed evidence published up to November 2020, and none of the previous reviews included all relevant publications on the latest advancements in treatment modalities or offered a comprehensive outcome analysis. Moreover, previous systematic reviews reported a need for well-conducted RCTs to provide robust evidence for clinical practice [24,25].

Therefore, this systematic review was designed to address the following PICO question: In adult patients with HCC [P], what is the efficacy and safety of TACE combined with I-125 brachytherapy [I] compared with TACE monotherapy [C] on OS, tumor response, PFS, and adverse events [O]. To reflect current practice, we did not exclude studies in which systemic therapies (e.g., sorafenib) were administered to both treatment arms, provided the only systematic difference between groups was the addition of I-125 brachytherapy. However, to isolate the specific effect of I-125 brachytherapy and maintain interpretability, we performed subgroup analyses stratifying by concurrent systemic therapy use and conducted sensitivity analyses excluding trials where systemic therapy was administered to either arm. We intended to critically appraise the effectiveness and safety profiles of these two treatment modalities, facilitating informed decision-making for clinicians to ensure the attainment of optimal patient outcomes and ultimately influencing future research to refine clinical guidelines for HCC management.

## 2. Methods

We followed the recommendations of the Cochrane Collaboration for this systematic review and meta-analysis [28] and reported the results according to the Preferred Reporting Items for Systematic Reviews and Meta-Analyses (PRISMA) guidelines [29] (Supplementary Material S1: PRISMA checklist). No generative AI programs were used in the preparation of this article. However, language enhancement and content structuring tools were utilized, specifically Grammarly [30] and Rubriq [31].

### 2.1. Protocol and Registration

The protocol of this systematic review and meta-analysis was predesigned by the research team and was registered in the PROSPERO database (ID: CRD42024516122) [32].

### 2.2. Search Strategy

We searched PubMed, EMBASE, the Cochrane Library, Scopus, and Web of Science for published studies and explored gray literature via Google Scholar, ProQuest, and ClinicalTrials.gov. The reference lists of the selected articles were also examined. The search covered 1 January 2010 to 30 November 2023, with filters for human studies and English-language publications. The search terms included hepatocellular carcinoma (HCC) and transarterial chemoembolization (TACE) and included Boolean operators, truncation, MeSH terms, and free-text keywords. A detailed search strategy is provided in Supplementary Material S2: Tables S1–S7.

### 2.3. Eligibility Criteria

Eligible studies met the following inclusion criteria:

- The population included adult patients (≥18 years old) diagnosed with HCC, and only studies with comparable baseline characteristics were included.
- Intervention: TACE combined with I-125 implantation (I-125 brachytherapy), with no limitations concerning agent, dose, methods, or duration of administration.
- Comparator: TACE monotherapy.
- Primary outcome: overall survival (after at least one year).
- The secondary outcomes included progression-free survival, tumor response rate, and safety.
- Study design: RCTs and non-randomized comparative studies (i.e., non-RCTs, case–control studies, or cohort studies) were considered.
- Language: English.

Studies were excluded if:

- The study focused on non-HCC patients.
- Patients diagnosed with multiple cancers were included.
- Different brachytherapy isotopes were used.
- The authors did not report any of the specified efficacy or safety outcomes.
- The design was non-comparative single-arm, or the studies were case series, case reports, abstracts, reviews, commentaries, or animal studies.

### 2.4. Search and Selection Process

The search and selection processes were conducted using Covidence software [33]. The titles and abstracts of all identified studies were independently screened by two reviewers against the inclusion criteria. Disagreements were resolved through discussion, and the final decision was made by a third reviewer. Following the same method, full-text articles were retrieved and assessed for eligibility.

### 2.5. Data Extraction and Quality Assessment

Two reviewers independently and systematically extracted data from the included studies via a designed data extraction form developed in Covidence software. The extracted data included study characteristics, patient demographics, tumor characteristics, descriptions of the control and intervention (e.g., chemotherapeutic agents, doses, and techniques), and primary and secondary outcomes. Disagreements were resolved through discussion, and the final decision was made by a third reviewer. We assessed the methodological quality of the included studies using two validated tools: the risk of bias in randomized controlled trials was assessed using the Cochrane Collaboration’s ROB-2 tool [34], whereas the quality of non-randomized studies was evaluated using the ROBINS-I tool [35].

### 2.6. Outcome

The primary outcome was overall survival (OS) at 1, 2, or 3 years. Secondary outcomes included at least one of the following efficacy outcome measures reported after at least one year: progression-free survival and tumor response rate assessed by modified RECIST criteria, which include complete response (CR), partial response (PR), stable disease (SD), and progressive disease (PD) [36]. Safety outcomes included at least one of the following: serious adverse events, treatment-related mortality, or liver function impairment.

### 2.7. Data Synthesis and Statistical Analysis

#### 2.7.1. Data Synthesis and Effect Measures

We conducted a meta-analysis via Stata 12.0 (Stata Corporation, College Station, TX, USA), RStudio version 2025.09.2 (Build 418; Posit Software, PBC), and Jamovi software (Version 2.6) to estimate pooled effect sizes for primary and secondary outcomes and compare them between the two groups. All meta-analyses for dichotomous outcomes (OS, tumor response, adverse events) were performed using the log Odds Ratio (log OR). The pooled log OR and its 95% confidence interval (CI) were then exponentiated and were reported as an odds ratio (OR) for ease of clinical interpretation. While Hazard Ratios (HRs) are the preferred metric for time-to-event data, the included studies predominantly reported survival as fixed-time proportions (e.g., 1-year OS rate). As individual patient data or full Kaplan–Meier curve data required for HR reconstruction were not available, ORs were selected as the most appropriate effect measure for the available data. Nevertheless, we note to our readers that ORs at fixed time points can be sensitive to baseline risk and variations in follow-up structure between studies; therefore, they do not fully depict the full survival curve. Hence, rather than being precise time-to-event measurements, these estimates should be considered approximations of treatment effect.

#### 2.7.2. Assessment of Heterogeneity and Model Selection

Statistical heterogeneity was quantified using Cochran’s Q-test (*p* < 0.1 indicating significant heterogeneity) and the I^2^ statistic, where I^2^ > 50% and *p* < 0.1 indicate substantial heterogeneity. Based on the I^2^ results, we selected appropriate models (fixed- or random-effects). All pooled analyses were conducted using a random-effects model, specified a priori, with variance components estimated using the DerSimonian–Laird estimator method to account for anticipated clinical and methodological heterogeneity across the included studies. For the secondary outcomes, results from the fixed-effects model were reported when there was a low level of heterogeneity, as they did not differ meaningfully from those obtained using the random-effects model. Subgroup and sensitivity analyses further explored heterogeneity. The Z test was used to evaluate the significance of the pooled outcome results (*p* < 0.05 was considered statistically significant).

#### 2.7.3. Management of Sparse Data and Assessment of Robustness

To enable odds ratio calculation for studies with zero events in one arm, a continuity correction of 0.5 was applied to all cells of the 2 × 2 table. Further, to verify the robustness of our findings, sensitivity analyses were performed using alternative methods (Peto odds ratio and treatment-arm continuity correction). All approaches yielded consistent results.

A comprehensive sensitivity analysis was conducted to assess the robustness of the findings: 1. leave-one-out analysis, which involved sequentially excluding each study to identify influential studies; 2. model comparison, namely fixed-effects vs. random-effects models; 3. study design, which involved excluding non-retrospective studies (*n* = 2); and 4. comparator consistency, which involved excluding studies with systemic therapy in control arms (*n* = 3). Studentized residuals and Cook’s distances identified potential outliers and influential studies.

#### 2.7.4. Subgroup Analysis and Investigation of Heterogeneity

We employed two complementary strategies to offer the most precise evaluation of our primary research question, which aims for a pure comparison of TACE + I-125 brachytherapy against TACE monotherapy. First, we performed subgroup analyses based on use of concurrent systemic therapy, study design (retrospective vs. prospective), and I-125 brachytherapy dosage [high-dose (≥100 Gy) and low-dose (<80 Gy) groups]. Second, a sensitivity analysis excluded studies where the control arm received systemic therapy (*n* = 3). This allowed assessment of whether I-125 brachytherapy benefits are independent of systemic treatment effects. Similarly, a highly restricted exclusion sensitivity analysis was conducted to provide the most rigorous assessment under the original research/PICO definition (TACE + I-125 brachytherapy vs. TACE alone). Sensitivity analyses included model comparison (fixed- vs. random-effects), leave-one-out analysis, and exclusion of studies where the control arm included systemic therapy.

Notably, due to incomplete reporting across studies and limited variability in most covariates, a meaningful meta-regression was not feasible for most outcomes. Also, analysis of the tumor size subgroup was not performed due to insufficient data availability, as the number of studies was insufficient to produce a statistically reliable result.

#### 2.7.5. Assessment of Publication Bias

The potential for publication bias was assessed using funnel plots for k ≥ 10, which were visually inspected for asymmetry. This was supplemented with statistical verification using Begg’s rank correlation test [37] and Egger’s regression test [38] (*p* < 0.05 indicating significance). A complementary fail-safe N analysis, File Drawer Analysis, was used to quantify the robustness of the pooled effect estimate against potential unpublished negative results.

### 2.8. Grading of Recommendations Assessment, Development and Evaluation (GRADE)

We performed a GRADE assessment for all primary and secondary outcomes. We used the Cochrane Handbook as our guide and no specific software was used. The GRADE tabulated results provided a structured evaluation of the certainty of evidence including the following points: risk of bias, inconsistency, indirectness, imprecision, and publication bias [39]. Two reviewers (A.H. and D.E.) independently assessed the certainty of evidence for each outcome, with disagreements resolved through discussion and final decision from M.I.M.I.

## 3. Results

### 3.1. Study Selection

**Figure 1:**
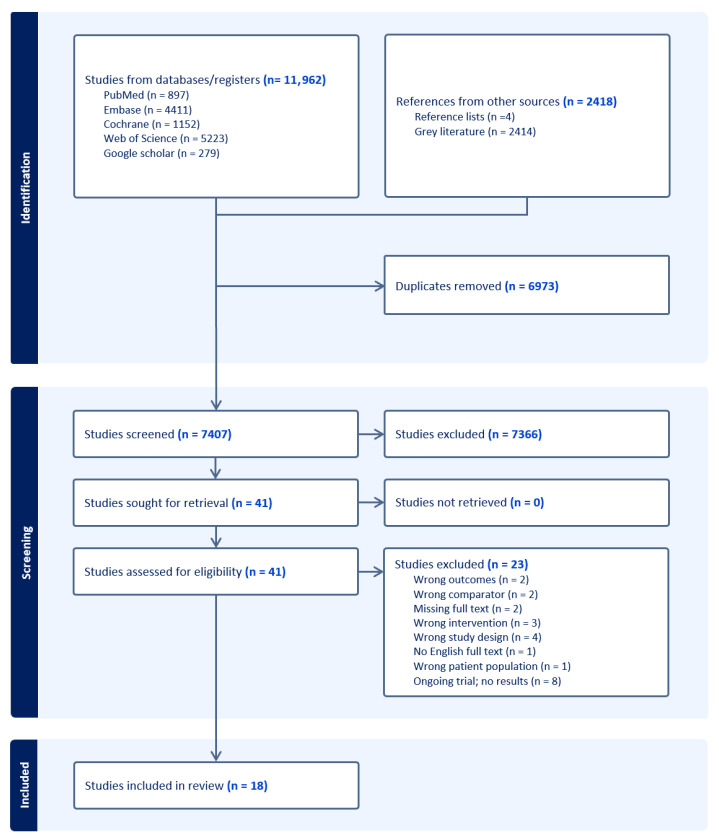
PRISMA flow diagram for systematic review record identification and selection [55].

A total of 14,380 records were identified from the searched databases. In total, 6973 records were identified as duplicates using Covidence and were removed. The remaining 7407 records were considered for title and abstract screening, and 7366 were excluded. Of the remaining 41 records screened for full text, 23 were excluded for the following reasons: irrelevant outcomes (*n* = 2), irrelevant comparators (*n* = 2), missing full texts (*n* = 2), irrelevant interventions (*n* = 3), unsuitable study designs (*n* = 4), no English full texts (*n* = 1), incorrect patient populations (*n* = 1), ongoing trials, and absence of results (*n* = 8). A detailed list of exclusion reasons for each study is included in Supplementary Material S3. The remaining studies (*n* = 18) were included in this systematic review [18,19,20,40,41,42,43,44,45,46,47,48,49,50,51,52,53,54]. The PRISMA flow diagram in Figure 1 summarizes the detailed inclusion/exclusion procedure.

### 3.2. Study Characteristics

Table 1 presents a summary of the characteristics of the 18 included studies [18,19,20,40,41,42,43,44,45,46,47,48,49,50,51,52,53,54]. Additional general details of these studies can be found in Table S8 in the Supplementary Material S2. All of the studies were published in China. One RCT and one non-RCT (non-randomized prospective/experimental study) were identified. The remaining 16 studies (*n* = 16) were retrospective cohort studies. The studies varied in terms of sample size, study design, patient characteristics, TACE protocols, chemotherapeutic agents used, and I-125 brachytherapy doses. Several treatment-specific variables were incompletely or inconsistently reported across studies. The I-125 brachytherapy radiation dose was unavailable in four studies, and among those reporting it, dosing ranges varied substantially. TACE drug or dose details were incompletely specified in two studies. Tumor burden metrics, including tumor size, were also inconsistently reported and missing in several studies. Consequently, subgroup analyses stratified by tumor burden, radiation dose, or chemotherapy regimen were feasible only among studies with complete reporting. 

### 3.3. Risk of Bias in the Included Studies

The risk of bias assessment was conducted using the ROBINS-1 risk of bias assessment tool for non-RCT studies (*n* = 17) and the RoB-2 tool for RCT studies (*n* = 1). Bias was assessed across seven domains using the ROBINS-I tool: D1—confounding, D2—participant selection, D3—intervention classification, D4—deviations from intended interventions, D5—missing data, D6—outcome measurement, and D7—selection of reported results. Assessment using the ROB-2 tool concentrated on five key areas of potential bias: confounding (D1), participant selection (D2), intervention classification (D3), deviations from intended interventions (D4), and missing data (D5).

**Figure 2:**
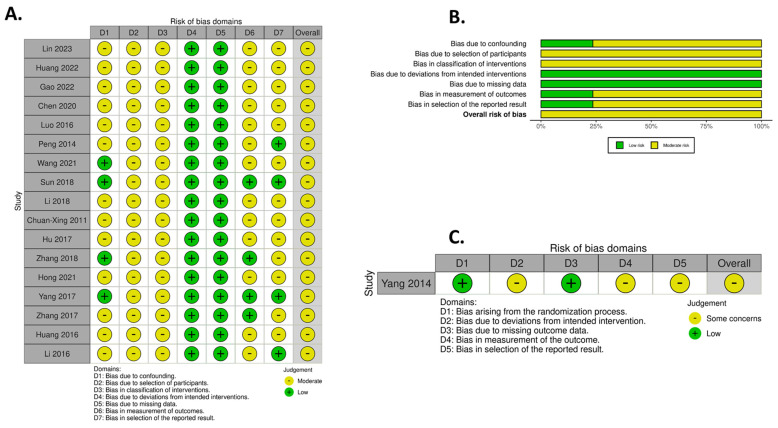
Risk of bias assessment results for the included studies. (**A**) ROBINS-1 risk of bias assessment result for each non-RCT clinical trial [18,19,40,41,42,43,44,45,46,47,48,49,50,51,52,53,54]; (**B**) overall ROBINS-1 risk of bias assessment results from all non-RCT clinical trials [18,19,40,41,42,43,44,45,46,47,48,49,50,51,52,53,54]; (**C**) ROB-2 risk of bias assessment result for the one RCT clinical trial included in this study [20]. The figures were generated via Robvis (https://mcguinlu.shinyapps.io/robvis/ (accessed on 6 March 2026)) web-based software [56].

Among the non-RCT studies, the ROBINS-1 assessment revealed that none of the studies were classified as having serious or critical risk of bias (Figure 2A). All were judged to have a moderate overall risk (Figure 2A). Notably, all studies showed low risk for deviations from intended interventions (D4) and missing data (D5) (Figure 2B).

The single randomized trial [20] was evaluated using the ROB-2 tool and was judged to have overall ‘some concerns’ (Figure 2C). It demonstrated low risk in the randomization process (D1) and for missing outcome data (D3). The overall judgment of ‘some concerns’ was driven by deviations from the intended interventions (D2), missing outcome data (D4), and selection of the reported result (D5).

### 3.4. Meta-Analysis of Outcomes

This section presents the meta-analysis results, including primary outcomes (OS at 1, 2, and 3 years) and secondary outcomes (ORR, DCR, PFS, and adverse events). All quantitative findings are summarized in Table S9 (Supplementary Material S2).

#### 3.4.1. Overall Survival (OS)

##### One-Year OS

**Figure 3:**
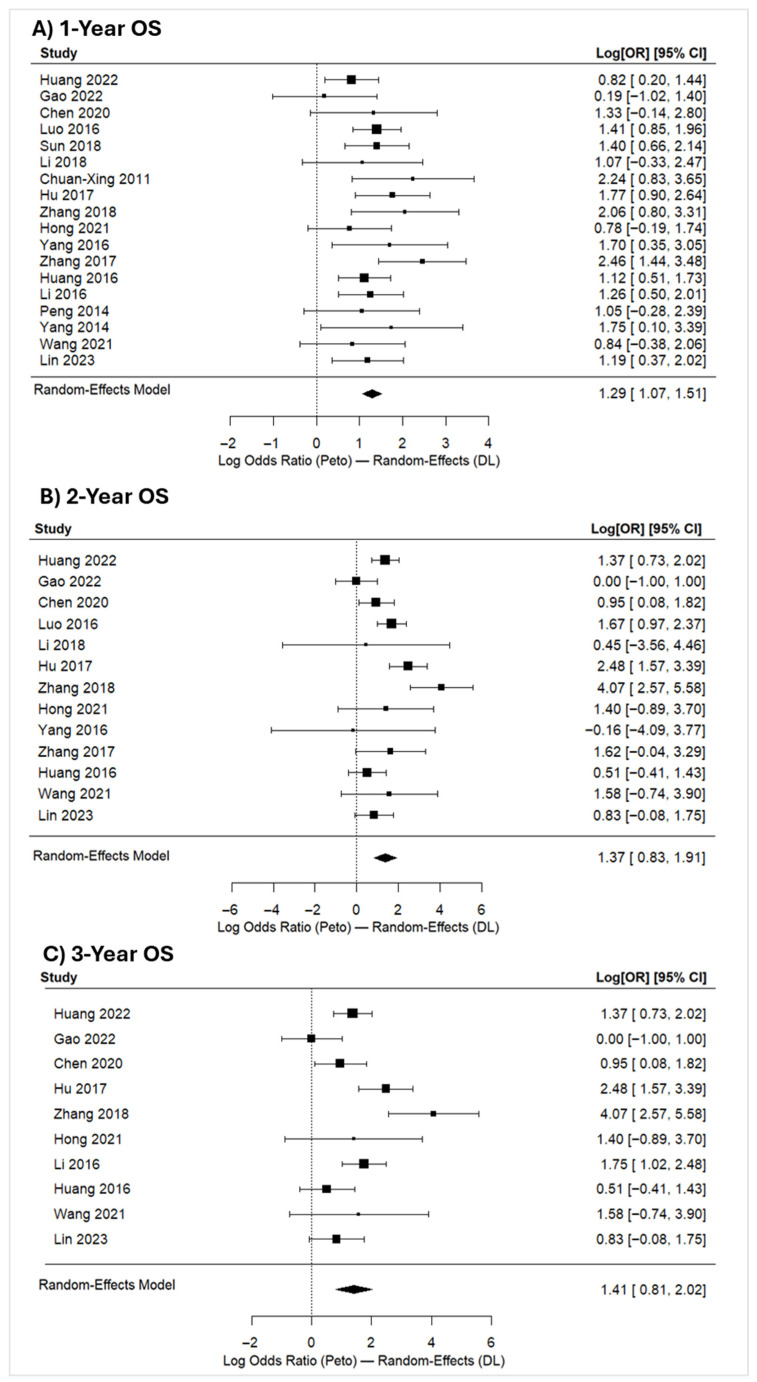
OS rates comparing TACE combined with iodine-125 brachytherapy versus TACE monotherapy in patients with HCC: (**A**) 1-year OS [18,19,20,40,41,42,43,44,45,46,47,48,49,50,51,52,53,54]; (**B**) 2-year OS [18,40,41,42,44,46,47,48,49,50,51,53,54]; (**C**) 3-year OS [18,40,41,42,44,45,48,50,53,54]. NB: Forest plots present pooled log odds ORs (Peto) with 95% confidence intervals calculated using a random-effects model (DerSimonian–Laird). Values > 0 favor the combination therapy (TACE + iodine-125) over TACE monotherapy.

A total of 18 studies were included (Figure 3A). Observed log odds ratios ranged from 0.1900 to 2.4589, with all estimates being positive. The estimated average log odds ratio from the random-effects model was 1.2929 (95% CI: 1.0710 to 1.5147), corresponding to an OR of approximately 3.64 (95% CI: 2.92–4.55). This represents 3.64-fold higher odds of 1-year survival with TACE + I-125 brachytherapy compared with TACE alone (z = 11.42, *p* < 0.001).

No significant heterogeneity was detected via the Q-test (Q(17) = 17.879, *p* = 0.397, I^2^ = 4.91%, tau^2^ = 0.01139). The low I^2^ value indicates high consistency across effect sizes, confirming that the pooled OR is a reliable representation of the overall treatment effect. Regarding model stability, analysis of the studentized residuals indicated that none of the studies exceeded the threshold of ±2.9913. Cook’s distances identified no overly influential outliers.

No publication bias was present. Both Begg’s rank correlation and Egger’s regression tests were non-significant (*p* = 0.3297; *p* = 0.4023, respectively), and the funnel plot showed no evidence of asymmetry (Supplementary Material S2: Figure S1).

Subgroup analyses based on study design revealed consistent benefits (Supplementary Material: Figure S2). For retrospective studies (k = 16), the pooled OR was 3.66 (95% CI: 2.87–4.66; *p* < 0.001), with low heterogeneity (I^2^ = 14.12%, tau^2^ = 0.03362, Q(15) = 17.466, *p* = 0.2918). For prospective studies (k = 2, including one RCT and one non-randomized prospective trial), the OR was 3.78 (95% CI: 1.34–10.67; *p* = 0.012); heterogeneity could not be reliably assessed due to the small number of studies. Subgroup analyses therefore revealed consistent benefits, but definitive conclusions about study design-specific effects were limited due to the small number of prospective studies. In addition, within each study, baseline characteristics were generally comparable between the arms, but the predominance of retrospective designs introduced potential selection bias that cannot be fully adjusted for in pooled analyses.

**Figure 4:**
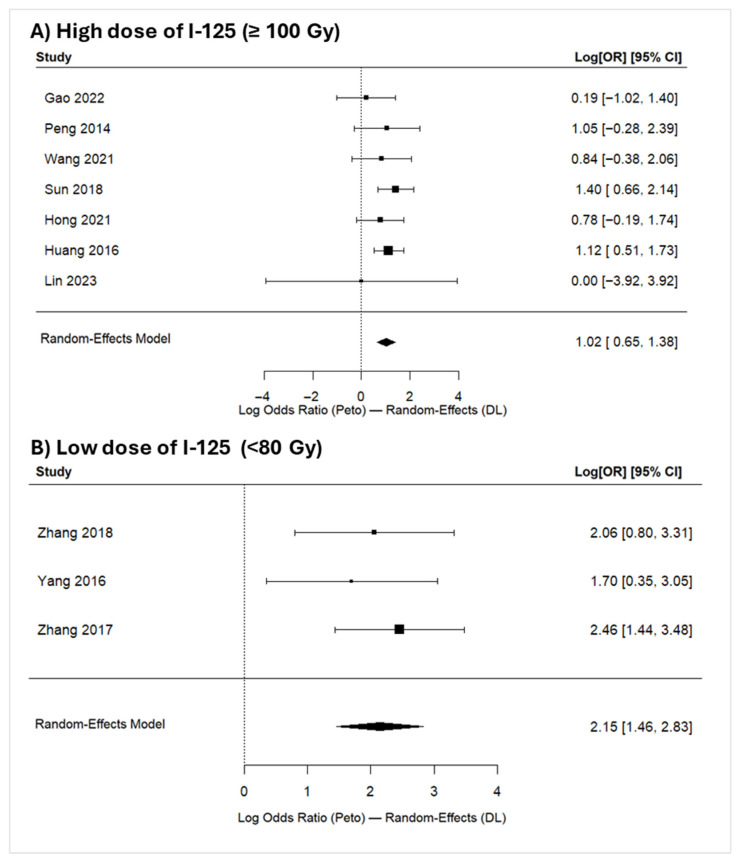
Comparison of 1-year OS between TACE combined with iodine-125 brachytherapy (low- vs. high-dose) and TACE monotherapy in patients with HCC. (**A**) High-dose of I-125 brachytherapy (≥100 Gy) [19,40,41,44,48,52,53]; (**B**) low-dose of I-125 brachytherapy (<80 Gy) [49,50,51]. NB: Forest plots present pooled log odds ORs (Peto) with 95% confidence intervals calculated using a random-effects model (DerSimonian–Laird). Values > 0 favor the combination therapy (TACE + iodine-125) over TACE monotherapy.

Subgroup analysis of 1-year OS by I-125 brachytherapy dose (high-dose [≥100 Gy, k = 7] vs. low-dose [<80 Gy, k = 3]) showed a significant benefit in both dose groups, with numerically larger effect estimates in the low-dose subgroup (Figure 4). The low-dose group’s estimated average log odds ratio was 2.1458 (95% CI: 1.4632 to 2.8284) yielding an OR of 8.55 (95% CI: 4.32–16.92; *p* < 0.001); however, heterogeneity could not be reliably assessed due to the limited number of studies. Conversely, the high-dose group showed an estimated average log odds ratio of 1.0531 (95% CI: 0.7196 to 1.3866); with an OR: 2.87 (95% CI: 2.05–4.00; *p* < 0.001) and no detectable heterogeneity (I^2^ = 0.00%, Q(6) = 3.414, *p* = 0.7554).

Furthermore, survival benefits were evaluated independently of concurrent systemic therapy (Supplementary Material S2: Figure S3). In studies without systemic therapy (k = 15), TACE + I-125 brachytherapy significantly improved 1-year survival compared to TACE alone (OR = 3.68, 95% CI: 2.89–4.70; *p* < 0.001). Similarly, in studies where both arms received systemic therapy (k = 3), the addition of I-125 brachytherapy was associated with higher 1-year survival (OR = 4.13, 95% CI: 1.69–10.09; *p* = 0.002). While substantial heterogeneity was observed in this subgroup (I^2^ = 72.56%, Q(2) = 7.29, *p* = 0.026), these estimates remain imprecise due to the small number of studies.

##### Two-Year OS

The analysis included 13 studies (Figure 3B), with observed log odds ratios ranging from −0.1597 to 4.0722; 85% were positive. The random-effects model yielded an estimated average log odds ratio of 1.3694 (95% CI: 0.8265 to 1.9124), corresponding to an OR of 3.93 (95% CI: 2.29–6.77). This average outcome differed significantly from zero (z = 4.9432, *p* < 0.0001), reinforcing the benefit seen at 1 year. The Q-test indicated significant heterogeneity (Q(12) = 32.4125, *p* = 0.0012, I^2^ = 62.98%), which is expected in longer-term survival analyses. While the random-effects model accounts for this variance, the level of heterogeneity suggests some divergence across individual study results. Outlier analysis via studentized residuals identified one study [50] exceeding the ±2.8905 threshold; however, Cook’s distance confirmed that no study exerted undue influence. Finally, no publication bias was observed, with non-significant rank correlation and regression tests (*p* = 0.7650 and *p* = 0.9546, respectively) and a symmetrical funnel plot (Supplementary Material S2: Figure S4).

##### Three-Year OS

Ten studies were included in this analysis (Figure 3C), with 90% of observed log odds ratios being positive (range: 0.0000 to 4.0722). The random-effects model yielded an average log odds ratio of 1.4147 (95% CI: 0.8061 to 2.0233), translating to an OR of 4.12 (95% CI: 2.24–7.56). This indicated that the odds of 3-year OS are about 4.12 times higher in the intervention group compared to the control group. The average outcome differed significantly from zero (z = 4.56, *p* < 0.0001), supporting a sustained long-term advantage for the combination therapy. The Q-test indicated significant heterogeneity (Q(9) = 31.9149, *p* = 0.002, I^2^ = 71.8000%); however, studentized residual analysis identified one potential outlier [50] exceeding ±2.8070, while Cook’s distance confirmed that no study was overly influential. Finally, rank correlation and regression tests showed no evidence of publication bias (*p* = 1.0000 and *p* = 0.4285, respectively; Supplementary Material S2: Figure S5).

#### 3.4.2. Cancer Response Rates

**Figure 5:**
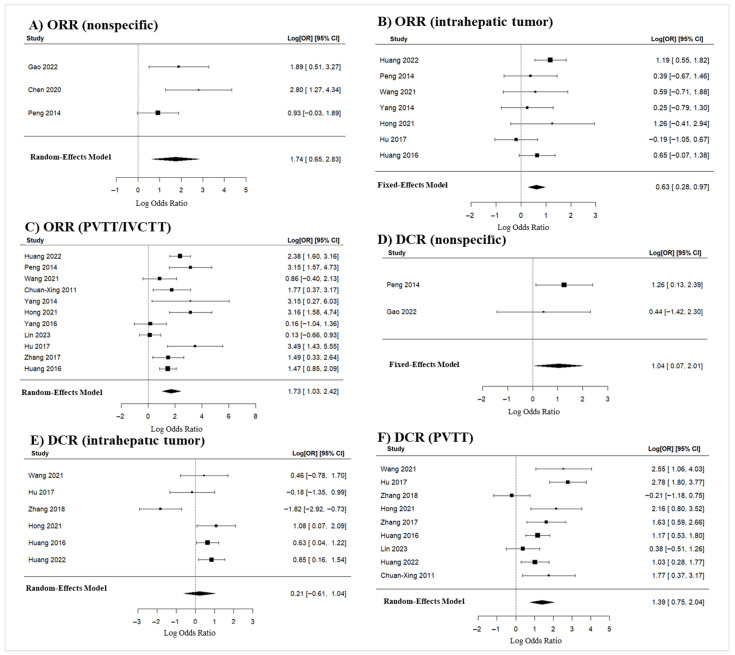
ORR and DCR comparing TACE combined with iodine-125 brachytherapy versus TACE monotherapy in patients with HCC. (**A**) Nonspecific [19,41,42]; (**B**) intrahepatic tumor [18,19,20,44,48,53,54]; (**C**) portal vein tumor thrombosis (PVTT) and/or inferior vena cava tumor thrombosis (IVCTT) [18,19,20,40,43,44,48,49,51,53,54]. DCRs for tumor location in the control and intervention groups; (**D**) Nonspecific [19,41]; (**E**) intrahepatic tumor [18,44,48,50,53,54]; (**F**) PVTT [18,40,43,44,48,50,51,53,54]. NB: Forest plots present pooled log ORs (Peto) with 95% confidence intervals. Values >0 favor TACE combined with iodine-125 brachytherapy over TACE monotherapy. Analyses were conducted using a random-effects model (DerSimonian–Laird), except for panels (**B**,**D**), which were analyzed using a fixed-effects model due to low heterogeneity (I^2^), with comparable results observed between models.

Tumor response outcomes for TACE plus I-125 brachytherapy versus TACE monotherapy are de-tailed in Table S10 (Supplementary Material S2). Response rates (i.e., CR, PR, SD, and PD) are reported as percentages, with the ORR and DCR compared via meta-analysis in Figure 5. The ORR was calculated as the sum of the CR and PR rates, whereas the DCR was calculated as the sum of the CR, PR, and SD rates.

##### Objective Response Rate (ORR)

ORR (Nonspecific)

Three studies reporting unspecified ORR (not specifically linked to intrahepatic tumor or PVTT) were included (Figure 5A). All observed log odds ratios were positive (range: 0.9282 to 2.8034). The random-effects model yielded an average log odds ratio of 1.7423 (95% CI: 0.6537 to 2.8309), translating to an OR of approximately 5.71 (95% CI: 1.92–16.96; *p* = 0.002). This result differed significantly from zero (z = 3.1369, *p* = 0.0017). Although the Q-test was non-significant (Q(2) = 4.41, *p* = 0.110), I^2^ (54.36%) and tau^2^ (0.503) indicated moderate heterogeneity, which should be interpreted cautiously given the small number of studies. The 95% prediction interval (−0.0237 to 3.5083) suggests that while the average outcome is positive, individual study outcomes may vary. Finally, studentized residuals (±(2.3940 threshold)) and Cook’s distances confirmed no influential studies or outliers.

ORR (Intrahepatic Tumor)

Seven studies were analyzed for intrahepatic tumor ORR (Figure 5B), with log odds ratios ranging from −0.1907 to 1.2629 (86% positive). The fixed-effects model yielded a significant average log odds ratio of 0.6255 (95% CI: 0.2830 to 0.9681; z = 3.5787, *p* = 0.0003), corresponding to 1.87 (95% CI: 1.33–2.63). No significant heterogeneity was detected (Q(6) = 7.7404, *p* = 0.2577, I^2^ = 22.4842%). All studentized residuals remained within ±2.6901 threshold, and Cook’s distances identified no disproportionately influential studies, confirming the absence of outliers.

ORR [Portal Vein Tumor Thrombosis (PVTT) or Inferior Vena Cava Tumor Thrombosis (IVCTT)]

Eleven studies were analyzed for ORR in patients with PVTT or IVCTT (Figure 5C), with all observed log odds ratios being positive (range: from 0.1313 to 3.4864). The random-effects model yielded an average log odds ratio of 1.7289 (95% CI: 1.0342 to 2.4236), translating to an OR of5.63 (95% CI: 2.81–11.29). This result differed significantly from zero (z = 4.8780, *p* < 0.0001), indicating a substantial benefit. Although the Q-test revealed significant heterogeneity (Q(10) = 35.4560, *p* = 0.0001; tau^2^ = 0.9151 and I^2^ = 73.9969%), studentized residuals remained within the ±2.8376 threshold, and Cook’s distances identified no studies with undue influence. The 95% prediction interval (−0.2705 to 3.7284) suggests that while the combined effect is strong, outcomes in individual future studies may vary. Finally, both rank correlation and regression tests revealed no evidence of publication bias (*p* = 0.2183 and *p* = 0.0513, respectively; Supplementary Material S2: Figure S6).

##### Disease Control Rate (DCR)

DCR (Nonspecific)

Two studies reporting unspecified DCR (not specifically linked to intrahepatic tumor or PVTT) were included (Figure 5D), with log odds ratios ranging from 0.4394 to 1.2615 (both positive). The fixed-effects model produced an average log odds ratio of 1.0390 (95% CI: 0.0712 to 2.0069), translating to an OR of 2.83 (95% CI: 1.07–7.45; *p* = 0.035). This differed significantly from zero (z = 2.1041, *p* = 0.0354). No heterogeneity was detected via the Q-test (Q(1) = 0.5471, *p* = 0.4595; I^2^ = 0.0000%); however, assessment is limited by the inclusion of two studies. Studentized residuals (with a ±2.2414 threshold) and Cook’s distances confirmed the absence of outliers or overly influential studies.

DCR (Intrahepatic Tumor)

Six studies were analyzed for intrahepatic tumor DCR (Figure 5E), with log odds ratios ranging from −1.8225 to 1.0788 (67% positive). The random-effects model yielded an average log odds ratio of 0.2137 (95% CI: −0.6123 to 1.0396), translating to an OR = 1.24 (95% CI: 0.54–2.83; *p* = 0.612). This difference was not statistically significant (z = 0.51, *p* = 0.612). Significant heterogeneity was detected via the Q-test (Q(5) = 20.7379, *p* = 0.0009, tau^2^ = 0.8205, I^2^ = 79.9367%), with a 95% prediction interval (−1.7444 to 2.1718) suggesting that true outcomes may vary across studies. Outlier analysis identified one study [50] exceeding the ±2.6383 studentized residual threshold; Cook’s distances confirmed that this study (Zhang et al., 2018) [50] was disproportionately influential.

DCR (PVTT/IVCTT)

Nine studies were analyzed for DCR in patients with PVTT or IVCTT (Figure 5F), with log odds ratios ranging from −0.2144 to 2.7820 (89% positive). The random-effects model yielded an average log odds ratio of 1.3941 (95% CI: 0.7496 to 2.0387), translating to an OR of 4.03 (95% CI: 2.12–7.68; *p* < 0.001). This average outcome differed significantly from zero (z = 4.2392, *p* < 0.0001). While the Q-test indicated significant heterogeneity (Q(8) = 28.0467, *p* = 0.0005, tau^2^ = 0.6869, I^2^ = 73.9507%), the 95% prediction interval (−0.3535 to 3.1417) suggests possible variability in individual study effects. Finally, studentized residuals (with a ±2.7729 threshold) and Cook’s distances confirmed the absence of outliers or overly influential studies.

#### 3.4.3. Progression-Free Survival (PFS)

The PFS outcomes are categorized by nonspecific PFS, intrahepatic tumor PFS, and PVTT PFS (Supplementary Material S2: Table S11). Because studies reported median PFS without interquartile ranges, a quantitative analysis of median values was conducted in lieu of meta-analysis. Across all categories, combination therapy consistently resulted in longer median PFS compared to TACE alone.

##### PFS (Nonspecific)

Three studies reported nonspecific PFS [41,42,47], consistently favoring TACE and I-125 brachytherapy over TACE alone. Reported medians included 11 vs. 5 months [41], 16 vs. 8 months [42], and 2.4 vs. 1.3 months [47]. In this category, the average median PFS was 13.5 months for combination therapy versus 6.5 months for TACE alone.

##### PFS (Intrahepatic Tumor)

Intrahepatic tumor PFS was reported only by Hong et al., who observed a median PFS of 5 months for the combination therapy with 2 months for TACE alone [53].

##### PFS (PVTT)

Three studies assessed PVTT PFS [40,43,53]. Reported medians included 7.9 vs. 5.3 months [43], 9 vs. 3 months [53], and 13 vs. 9 months [40]. The average median for this category was 9.97 months for combination therapy versus 5.77 months for TACE alone. Overall, these descriptive results consistently show that combining TACE with I-125 brachytherapy implantation extends PFS across all categories compared with TACE monotherapy.

#### 3.4.4. Meta-Analysis of Adverse Events and Complications

Safety outcomes for 11 adverse events revealed no significant differences between groups (Figure 6). Detailed forest plots and individual study outcomes for each adverse event are provided in the Supplementary Material S2 (Figures S7–S17).

No statistically significant differences were observed between the intervention and control groups across all evaluated adverse events. Publication bias assessment, conducted for nausea/vomiting, fever, and abdominal pain, showed no evidence of asymmetry (Supplementary Material S2: Figures S7–S9).

Gastrointestinal and General Symptoms

For nausea/vomiting (k = 14), the average log OR was −0.1256 (OR = 0.88; *p* = 0.6431), with considerable heterogeneity (I^2^ = 75.73%). Other side effects, including diarrhea (k = 3, OR = 0.99; *p* = 0.9688), fever (k = 14, OR = 1.08; *p* = 0.5295), and abdominal pain (k = 10, OR = 1.20; *p* = 0.2171), showed no significant group differences or heterogeneity.

Organ-Specific and Procedural Complications

Liver abnormalities (k = 5, OR = 0.95; *p* = 0.8792), GIT bleeding (k = 4, OR = 1.12; *p* = 0.7514), biloma (k = 2, OR = 1.57; *p* = 0.4661), and liver abscesses (k = 4, OR = 1.59; *p* = 0.5509) all demonstrated non-significant effects with no detectable heterogeneity.

Other Outcomes

Analyses of myelosuppression (k = 2, OR = 1.82; *p* = 0.1953), hypertension (k = 2, OR ≈ 0.87; *p* = 0.6592), and ascites (k = 2, OR = 0.84; *p* = 0.6035) further confirmed the comparable safety profile between treatments.

### 3.5. GRADE

The GRADE Summary of Findings table (Table 2) below presents the certainty of evidence for primary and key secondary outcomes. In addition, the detailed GRADE assessments are provided in Supplementary Material Table S12.

The certainty of evidence was low to very low across all outcomes (Supplementary Materials, Table S12), which significantly impacts how the findings should be interpreted: (a) 1-year overall survival: the GRADE rating is low, indicating that there is limited confidence in the finding and that the true effect may differ substantially from our estimate; (b) 2 and 3-Year Survival: GRADE rating is very low, indicating very little confidence and that the true effect is likely materially different from our pooled estimate; (c) ORR (PVTT/IVCTT): GRADE rating is very low, indicating high uncertainty due to serious inconsistency in the data; (d) Adverse Events: GRADE rating is very low, indicating high uncertainty due to serious imprecision. The factors that contributed to uncertainty are (1) the reliance on observational studies where the GRADE evidence base starts at a low certainty level by default for this particular study design; and (2) further downgrading due to significant variations in long-term outcomes and a lack of precision regarding side effects.

## 4. Discussion

This systematic review and meta-analysis explored the efficacy and safety of combining TACE with I-125 brachytherapy compared with TACE monotherapy in patients with HCC. The studies included in this analysis, published between 2011 and 2023, were mainly from China, with one RCT and one non-RCT clinical trial among multiple retrospective cohort studies. Our findings suggest that adding I-125 brachytherapy to TACE is associated with favorable tumor control outcomes and an acceptable safety profile in appropriately selected patients. This approach significantly increased overall survival rates (at 1, 2, and 3 years), ORR, and DCR, particularly for intrahepatic tumors and PVTT/IVCTT, without increasing major adverse events.

The relative effectiveness of TACE in improving OS in patients with HCC has been investigated in numerous studies [14,41,57,58,59,60,61]. While TACE, alone or in combination, enhances tumor response and quality of life, OS advantages for TACE monotherapy are limited [62,63]. Notably, combinations of TACE with other modalities (e.g., additional locoregional or systemic approaches) more consistently improve both OS and PFS compared with TACE alone [14,58,59,61,64].

The integration of I-125 seeds, which deliver continuous localized radiation, supports prolonged tumor remission and contributes to the observed survival benefits [21]. Notably, some observational studies have suggested that, in selected patient cohorts, TACE + I-125 brachytherapy may offer survival outcomes that compare favorably with those of sorafenib-based regimens [65], these indirect comparisons are highly confounded and should not be interpreted as evidence of superiority. Well-conducted head-to-head randomized trials are required to confirm these findings. Only such evidence would support the potential of TACE with I-125 brachytherapy as a superior intervention (over established systemic therapies) for advanced HCC patients with vascular invasion, specifically PVTT.

### 4.1. Meta-Analysis Findings

#### 4.1.1. Overall Survival (OS)

Our meta-analysis of OS at 1, 2, and 3 years demonstrated significant survival benefits for patients receiving TACE + I-125 brachytherapy, with patients having approximately 3.64, 3.93, and 4.12 times the odds of survival compared to TACE alone at 1, 2, and 3 years, respectively. Other publications examining variable doses of I-125 brachytherapy in different interventions have reported effectiveness using various I-125 brachytherapy doses [50,66,67]. Although not compared head-to-head, the subgroup analysis of studies using high-doses of I-125 brachytherapy (≥100 Gy) versus those using lower doses (<80 Gy) indicated that both dose categories were associated with a significant 1-year overall survival benefit for TACE plus I-125 brachytherapy relative to TACE alone. While a higher point estimate was observed in the low-dose subgroup, this subgroup included only three studies; therefore, this result should be interpreted cautiously and considered hypothesis-generating rather than indicative of a true dose–response relationship. 

#### 4.1.2. ORR and DCR

Meta-analyses of the ORR and DCR provided critical insights into the efficacy of interventions across tumor subtypes. For nonspecific tumors, the significantly positive ORR and DCR suggest the broad effectiveness of the interventions. Intrahepatic tumor analyses revealed a moderate but significant ORR and a nonsignificant DCR, reflecting variability in treatment response. The lack of significance in DCR could indicate either variability in the intervention’s efficacy or differences in study design and population characteristics, highlighting the need for standardized protocols in future studies. For PVTT/IVCTT tumors, the strong, significantly positive ORR and DCR, despite heterogeneity, reinforce the potential efficacy of the intervention, especially in advanced disease. However, previous meta-analyses have produced contradictory results regarding the ORR for PVTT tumors and have not reported any outcomes on the DCR [24,68].

#### 4.1.3. Progression-Free Survival (PFS)

PFS outcomes varied across tumor types, with the most notable improvements observed in the PVTT subgroup. The clinical benefit of I-125 brachytherapy likely stems from its precise intra-tumoral placement, delivering sustained, localized radiation with minimal collateral damage to promote prolonged remission [21]. These findings are particularly important for PVTT, which is typically associated with poor prognosis and limited treatment options. Given its efficacy, including outperforming systemic agents in specific cohorts, TACE plus I-125 brachytherapy represents a potentially beneficial intervention for advanced HCC and vascular invasion.

#### 4.1.4. Safety Analysis

Safety remains paramount when combining therapies for advanced-stage cancers. Our analysis revealed no significant differences in adverse events between combination therapy and TACE monotherapy. The comparable incidence of side effects, such as nausea, vomiting, or diarrhea, aligns with existing literature and suggests that adding I-125 to TACE is well-tolerated and clinically feasible [53,69]. Notably, the localized delivery of I-125 brachytherapy may offer a safety advantage over systemic treatments by restricting radiation to the tumor area and minimizing damage to surrounding organs [21].

### 4.2. Strengths and Limitations

#### 4.2.1. Strengths

Previous meta-analyses on this topic, most recently published in 2022, included studies only up to November 2020 and smaller patient populations (351–1098 patients) [24,25,68,70,71]. In contrast, our analysis is more comprehensive, incorporating studies through 2023 and the largest cohort to date (*n* = 1872; 894 interventions, 930 control). We provide the most detailed safety assessment, evaluating 11 adverse events compared with 3–6 previously reported, with some earlier reviews lacking quantitative safety analyses altogether. Additionally, we are the first to report overall survival at 1, 2, and 3 years, offering a more granular evaluation of long-term outcomes, and to present the most extensive synthesis of tumor response metrics (CR, PR, SD, PD), including the first meta-analyses of ORR and DCR.

#### 4.2.2. Limitations

First, the dominance of retrospective cohort studies (16 of 18) in the evidence base, with only one non-randomized prospective trial and one RCT included, limits the strength of the conclusions. As a result, the pooled estimates are susceptible to both residual confounding and selection bias. Second, all 18 studies originated from China; this restricts generalizability to non-Asian populations, different HCC etiologies, and healthcare systems with different practice patterns. To illustrate further, several important implications need to be highlighted: (a) In China, hepatitis B virus infection is the predominant underlying cause (i.e., etiology) of HCC, whereas in Western populations, other causes account for a larger proportion of cases (e.g., hepatitis C, alcohol-related liver disease, and NAFLD); (b) Practice patterns, specifically TACE protocols and I-125 brachytherapy techniques implemented in the reported studies, can only reflect Chinese expertise and may not be directly transferable to other healthcare settings; (c) Patient characteristics in the reported studies may differ from those in non-Asian populations, including but not limited to the demographic profile, prevalence and severity of PVTT, and tumor burden. Consequently, the applicability of our findings to global clinical practice and guideline development can be limited. And we endorse the conduct of robust multicenter international trials to validate the findings across diverse populations. Third, the restriction to English-language publications can introduce bias. Fourth, this meta-analysis relied on the use of ORs for survival outcomes; while necessitated by the absence of individual patient data or Kaplan–Meier curves, this may carry the risk of overestimating treatment effects in the presence of high event rates. Fifth, considerable clinical heterogeneity was observed regarding tumor burden, TACE protocols, dosing of I-125 brachytherapy, and concurrent systemic therapies. Consequently, the pooled effect estimates represent an average effect across these diverse clinical contexts and may not apply uniformly to every patient subgroup. Sixth, for low-dose (<80 Gy) I-125 brachytherapy, the apparently larger effect estimate should be interpreted with caution and considered hypothesis-generating rather than solid evidence of a true dose–response relationship. Finally, the certainty of evidence (GRADE framework) was low to very low across all outcomes, which significantly impacts how the findings should be interpreted.

### 4.3. Future Perspectives

Future research should prioritize well-designed, international, multicenter RCTs with standardized protocols to validate survival benefits. Additionally, the long-term safety of I-125 brachytherapy, particularly with respect to radiation-induced liver toxicity, remains a key concern. Further research should optimize I-125 brachytherapy dosage, spacing, and deployment using advanced imaging techniques, such as CT- or MRI-guided navigation. Investigating novel therapeutic combinations, such as immune checkpoint inhibitors with TACE and I-125 brachytherapy, may reveal synergistic effects that enhance treatment efficacy in advanced HCC patients. Research should also include multiethnic cohorts to improve generalizability. Conducting prospective, multicenter RCTs with standardized outcome measures is crucial for reducing variability and ensuring reproducibility. Rigorous methodologies, including stratified randomization and standardized reporting, are essential for mitigating biases and translating findings into clinical practice.

### 4.4. Implications for Clinical Practice

This review highlights the clinical impact of combining I-125 brachytherapy with TACE for advanced HCC, particularly in patients with PVTT, for whom the prognosis is poor. By delivering sustained, localized radiation, this approach may be associated with enhanced tumor control and survival beyond TACE alone, preventing aggressive tumor behavior and vascular invasion. Given the demonstrated efficacy and safety profile, this combination therapy has the potential to be integrated into standard treatment guidelines, offering a personalized option for patients ineligible for surgery or transplantation. Nevertheless, it is important to recognize that the included studies varied considerably in tumor burden, TACE protocol, dosing of I-125 brachytherapy, and use of concomitant systemic treatments. Therefore, the findings represent an average effect across diverse clinical scenarios and may not uniformly apply to every patient subgroup. We recommend that clinicians recognize this before extrapolating these meta-analysis findings indiscriminately to HCC populations that were not represented. We also note that this may limit the applicability of the findings to guideline development outside of China.

## 5. Conclusions

This systematic review and meta-analysis evaluated TACE combined with I-125 brachytherapy for advanced HCC, predominantly in patients with PVTT. Compared with TACE alone, the combination therapy was associated with significantly improved overall survival at 1, 2, and 3 years and improved tumor response rates, without a statistically significant increase in major adverse events. However, GRADE assessment indicated low certainty for 1-year overall survival and very low certainty for longer-term survival, tumor response, and adverse events. Subgroup analyses suggested a potential dose–response pattern, with studies using lower I-125 brachytherapy doses (<80 Gy) showing higher point estimates for survival benefit than those using higher doses; however, this finding should be interpreted cautiously given the limited number of studies in some subgroups and should be confirmed by adequately powered RCTs. The combination therapy also improved tumor response outcomes, including ORR and DCR, particularly among patients with vascular invasion. Overall, the available evidence, based predominantly on retrospective Chinese cohorts, indicates that TACE combined with I-125 brachytherapy may enhance local tumor control and delay disease progression, representing a promising therapeutic option for high-risk patients with advanced HCC. Future studies with robust study designs in diverse populations with standardized dosing protocols are highly warranted to establish definitive clinical recommendations and optimize therapeutic outcomes.

## Figures and Tables

**Figure 6:**
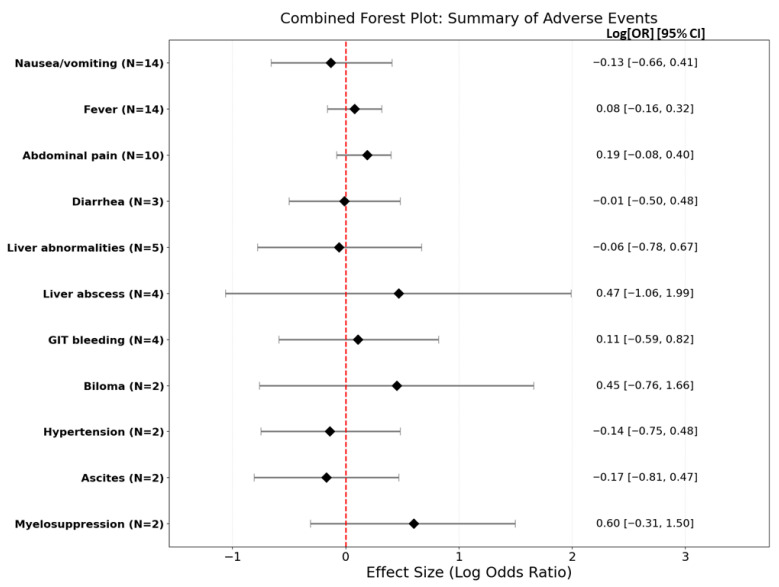
Pooled analysis of adverse events comparing TACE combined with iodine-125 brachytherapy versus TACE monotherapy in patients with HCC. Forest plot showing the summary pooled log odds ratios and 95% CIs for 11 reported adverse events in the control and intervention groups. The diamond markers represent pooled estimates, and the vertical dashed line indicates no effect. The red dashed line also indicates no effect. See Supplementary Material (Figures S7–S17) for full forest plots containing individual study data per adverse event [18,19,20,40,41,42,43,44,45,46,47,48,49,50,51,52,53,54]. NB: Analyses were conducted using a fixed-effects model, except for nausea/vomiting, which was analyzed using a random-effects model due to high heterogeneity (I^2^), with comparable results observed across both models.

**Table 1:** Baseline characteristics of the patients in the included studies, their index tumor characteristics, and their arms.

	Author and Year	Patients (N)	Gender (M\F)	Age (Mean ± SD, Years)	Tumor Size (Mean ± SD, cm)	No. of Tumors	Child–Pugh Class (A/B/C)	Etiology (Hep B\Hep C\Other)	I-125 Dose (Gy)	TACE Treatment Drug and Dose
1	Huang 2021 * [18]									
	Control	97	86\11	51 ± 12.0	7.5 ± 2.4	1\≥2:34\63	70\27\0	84\8\5		TACE: Epirubicin (dose NR); Systemic: Sorafenib 400 mg BID
	Intervention	74	68\6	49 ± 12.5	6.8 ± 2.0	1\≥2:23\51	56\18\0	65\5\4	NR	AA
2	Gao 2022 [41]									
	Control	32	26\6	62.1 ± 13.3	5.8 ± 2.7	1\≥2:19\12	25\7\0	31\0\1		5-fuorouracil (150 mg), mitomycin C (10 mg), epirubicin (50 mg)
	Intervention	32	26\6	62.7 ± 11.8	5.5 ± 1.9	1\≥2:25\7	26\6\0	22\0\10	100–140	AA
3	Chen 2020 [42].									
	Control	48	38\10	59.6 ± 10.1	<3\≥3:13\35	1\≥2:24\24	35\13\0	27\0\21		Doxorubicin (10–20 mg)
	Intervention	35	26\9	58.1 ± 10.1	<3\≥3:14\21	1\≥2:16\19	24\11\0	18\0\17	90–165	AA
4	Luo 2015 [47]									
	Control	94	82\12	55.1 ± 11.1	>5\ ≤5:61\33	NR	86\8\0	74/10/10		Epirubicin (10–50 mg)
	Intervention	182	167\15	53.6 ± 10.2	>5\ ≤5:123\59	NR	160\22\0	154/16/12	37–180.7	AA
5	Peng 2014 [19]									
	Control	43	39\4	≥50\<50:21\22	NR	1–3\>3:26\17	38\5\0	31\1\11		lobaplatin (10–50 mg)
	Intervention	32	31\1	≥50\<50:17\15	NR	1–3\>3:15\17	27\5\0	23\0\9	120	AA
6	Wang 2021 [48]									
	Control	25	23\2	<50\≥50:12\13	<5\≥5:5\20	NR	19\6\0	19\0\6		Pirarubicin (20–40 mg)
	Intervention	21	18\3	<50\≥50:11\10	<5\≥5:6\15	NR	15\6\0	18\0\3	100	AA
7	Sun 2018 [52]									
	Control	70	58\12	55\≥55:39\31	5\>5:15\55	NR	31\39\0	60\4\6		Pirarubicin (30–40 mg), floxuridine (750–1000 mg), mitomycin (10 mg).
	Intervention	64	45\19	55\≥55:30\34	5\>5:18\46	NR	25\39\0	49\6\9	100–120	AA
8	Li 2018 [46]									
	Control	33	25\8	54.64 ± 11.58	4.763 ± 1.501	NR	7\26\0	22\4\7		Doxorubicin (20–40 mg)
	Intervention	21	17\4	56.14 ± 9.82	4.809 ± 1.571	NR	4\17\0	14\3\4	NR	AA
9	Chuan-Xing 2011 [43]									
	Control	30	7\23	51 ± 2.3	NR	NR	17\13\0	21\0\9		Oxaliplatin (135 mg), epirubicin (30–40 mg)
	Intervention	26	9\17	48 ± 1.6	NR	NR	13\11\0	23\0\3	>40	AA
10	Yang 2013 [20]									
	Control	42	39\3	≥50\<50:23\19	≥5\<5:29\13	NR	23\19\0	40\1\1		Sorafenib (50–75 mg)
	Intervention	43	39\4	≥50\<50:25\18	≥5\<5:28\15	NR	24\19\0	40\2\1	NR	AA
11	Hu 2017 [54]									
	Control	50	40\10	45.4 ± 5.2	>5\≤5:31\19	NR	44\6\0	40\8\2		Doxorubicin (20–40 mg)
	Intervention	50	42\8	47.6 ± 6.3	>5\≤5:30\20	NR	42\8\0	42\7\1	NR	AA
12	Zhang 2018 [50]									
	Control	56	48\8	≥55\<55:34\22	≥10\<10:17\39	NR	54\2\0	56\0\0		Epirubicin (10–50 mg)
	Intervention	20	19\1	≥55\<55:11\9	≥10\<10:5\15	NR	18\2\0	20\0\0	58.3–64.0	AA
13	Hong 2021 [53]									
	Control	35	25\10	54.5 ± 8.4	8.7 ± 2.5	Single\multiple: 20\14	32\3\0	33\1\1		Epirubicin (40 mg)
	Intervention	34	29\5	58.1 ± 7.3	7.6 ± 3.0	Single\multiple: 17\18	33\1\0	31\2\1	120	AA
14	Yang 2016 [49]									
	Control	28	25\3	50.86 ± 12.116	≥10\<10:13\15	NR	20\8\0	21\2\5		Epirubicin (10–50 mg)
	Intervention	33	27\6	53.30 ± 8.640	≥10\<10:13\20	NR	22\11\0	22\2\9	60.6–76.6	AA
15	Zhang 2017 * [51]									
	Control	31	26\5	≥55\<55:14\17	≥5\<5:19\12	NR	24\7\0	29\0\2		TACE: Epirubicin (10–50 mg); Systemic: Sorafenib (400 mg BID)
	Intervention	37	34\3	≥55\<55:18\19	≥5\<5:26\11	NR	33\4\0	35\0\2	57.4–65.3	AA
16	Huang 2016 [44]									
	Control	140	127\13	51.6 ± 10.8	<7\≥7:83\57	<3/≥3:70/70	68\72\0	140\0\0		Doxorubicin (20–60 mg), lobaplatin (50 mg)
	Intervention	70	63\7	51.1 ± 11.1	<7\≥7:39\31	<3/≥3:30/40	31\39\0	70\0\0	120	AA
17	Li 2016 [45]									
	Control	78	67\11	48.1 ± 10.0	4.00 ± 0.55	1\2\3–4:26\39\13	63\15\0	60\0\18		Pirarubicin (20 mg) and cisplatin (50 mg)
	Intervention	66	56\10	48.8 ± 10.7	3.97 ± 0.58	1\2\3–4:21\33\12	58\8\0	62\0\4	90–165	AA
18	Lin 2023 * [40]									
	Control	45	42\3	57.0 ± 6.4	9.0 ± 3.7	≤33/>3:3/42	(5–7)/(7–9):28/17	42\0\3		TACE: drug name NR; Systemic: Lenvatinib (4–12 mg PO) and camrelizumab (200 mg IV)
	Intervention	55	48\7	54.2 ± 11.7	8.6 ± 3.6	≤33/>3:5/50	(5–7)/(7–9):33/32	50\0\5	110–140	AA

* Studies that included systemic therapy in both arms in addition to TACE. AA: as above; BID: twice per day; IV: intravenous; N: number; NR: not reported; PO: oral; TACE: transarterial chemoembolization; I 125: iodine-125 brachytherapy.

**Table 2:** Summary of GRADE Findings.

Outcome	Certainty Rating	Justification for Rating
1-year overall survival	⨁⨁◯◯ LOW	Retrospective cohort studies (start at LOW). No further downgrading: - Low inconsistency (I^2^ = 4.9%). - No serious imprecision (95% CI excludes null, OIS met). - No publication bias detected.
2-year overall survival	⨁◯◯◯ VERY LOW	Retrospective cohort studies (start at LOW). Downgraded one level: serious limitations in study design. Downgraded one level: serious inconsistency (I^2^ = 63.0%).
3-year overall survival	⨁◯◯◯ VERY LOW	Retrospective cohort studies (start at LOW). Downgraded one level: serious limitations in study design. Downgraded one level: serious inconsistency (I^2^ = 71.8%).
ORR (PVTT/IVCTT)	⨁◯◯◯ VERY LOW	Retrospective cohort studies (start at LOW). Downgraded one level: serious limitations in study design. Downgraded one level: serious inconsistency (I^2^ = 74.0%).
Adverse events (any)	⨁◯◯◯ VERY LOW	Retrospective cohort studies (starting LOW). Downgraded one level: serious limitations in study design. Downgraded one level: serious inconsistency (e.g., nausea/vomiting I^2^ = 75.7%). Downgraded one level: serious imprecision (CIs cross null and clinically important thresholds).

NB: (1) The direction of effect is consistent, but the certainty of evidence is limited, and results should be interpreted with caution. (2) No outcomes qualified for upgrading despite large effect sizes (ORs > 2.0) since upgrading was precluded by the presence of downgrading in other domains.

## Data Availability

All the data generated or analyzed during this study are included in this article and its Supplementary Material files. Further details are available from the corresponding authors upon reasonable request.

## Supplementary Materials

The following supporting information can be downloaded at: https://www.mdpi.com/article/10.3390/jcm15062267/s1, The supplementary materials include detailed database search strategies, baseline characteristics of the included studies, characteristics of the included studies, PRISMA checklist, Study Exclusion Reasons, risk-of-bias assessment tables, and additional analyses (subgroup, sensitivity, and forest plots) that support the findings of this systematic review and meta-analysis. Table S1: PubMed database search strategy and results; Table S2: Embase database search strategy and results; Table S3: Cochrane database search strategy and results; Table S4: Google scholar database search strategy and results; Table S5: Web of Science database search strategy and results; Table S6: Proquest database search strategy and results; Table S7: clinicaltrials.gov search strategy and results; Table S8: General details on the included studies; Table S9: Summary of Meta-Analysis Results (TACE + I-125 brachytherapy vs. TACE Monotherapy); Table S10: Comparison of tumor response between the intervention group (TACE plus I-125 brachytherapy) versus the control group (TACE monotherapy) in patients with hepatocellular carcinoma; Table S11: PFS outcome (median months); Table S12: Summary of Grading of Recommendations Assessment, Development and Evaluation (GRADE) Findings; Figure S1: Regression Test for Funnel Plot Asymmetry_OS at 1 year from all included studies; Figure S2: Comparison of the 1-year OS rates between the control and intervention groups. (a) Retrospective studies only; (b) Prospective studies only; Figure S3: Comparison of the 1-year OS rates between the control and intervention groups. (a) Studies without systemic chemotherapy; (b) Studies with systemic chemotherapy; Figure S4: Regression Test for Funnel Plot Asymmetry_OS at 2 year from all included studies; Figure S5: Regression Test for Funnel Plot Asymmetry_OS at 3 year from all included studies; Figure S6: Regression Test for Funnel Plot Asymmetry_ PVTT/IVCTT ORR; Figure S7: (A) Forest plot of log odds ratios with 95% confidence intervals for the analysis of nausea and vomiting in the intervention versus control groups. (B) Regression test for funnel plot asymmetry among studies included in the nausea and vomiting outcome analysis. Figure S8: (A) Forest plot of log odds ratios with 95% confidence intervals for the analysis of fever in the intervention versus control groups. (B) Regression test for funnel plot asymmetry among studies included in the fever outcome analysis. Figure S9: (A) Forest plot of log odds ratios with 95% confidence intervals for the analysis of abdominal pain in the intervention versus control groups. (B) Regression test for funnel plot asymmetry among studies included in the abdominal pain outcome analysis. Figure S10: Forest plot of log odds ratios with 95% confidence intervals for the analysis of diarrhea in the intervention versus control groups. Figure S11: Forest plot of log odds ratios with 95% confidence intervals for the analysis of liver abnormalities in the intervention versus control groups. Figure S12: Forest plot of log odds ratios with 95% confidence intervals for the analysis of liver abscess in the intervention versus control groups. Figure S13: Forest plot of log odds ratios with 95% confidence intervals for the analysis of GIT bleeding in the intervention versus control groups. Figure S14: Forest plot of log odds ratios with 95% confidence intervals for the analysis of biloma in the intervention versus control groups. Figure S15: Forest plot of log odds ratios with 95% confidence intervals for the analysis of hypertension in the intervention versus control groups. Figure S16: Forest plot of log odds ratios with 95% confidence intervals for the analysis of ascites in the intervention versus control groups. Figure S17: Forest plot of log odds ratios with 95% confidence intervals for the analysis of myelosuppression in the intervention versus control groups. Supplementary Material S3: Study Exclusion Reasons.
