# Supplementary material for: Evaluating the Efficacy and Safety of TACE Combined with Iodine-125 Brachytherapy Versus TACE Monotherapy for Hepatocellular Carcinoma: A Systematic Review and Meta-Analysis
Source: J Clin Med. 2026 Mar 17;15(6):2267. doi: 10.3390/jcm15062267 (PMC13026359; doi:10.3390/jcm15062267)
Supplement: Supplementary file 1 [file jcm-15-02267-s001.zip › jcm-4148033 -Supplementary_TACE MA_Updated Mar 5.pdf]

Main databases search

Table S1:PubMed database search strategy and results

**Database**	Pubmed
**Access date**	01-Dec-2023
**Filters applied**	Clinical Trial, Comparative Study, Observational Study, Randomized Controlled Trial, Humans, English.
**Search strategies**	("carcinoma, hepatocellular"[MeSH Terms] OR ("carcinoma"[Title/Abstract] AND "hepatocellular"[Title/Abstract]) OR "hepatocellular carcinoma"[Title/Abstract] OR ("hepatocellular"[Title/Abstract] AND "carcinoma"[Title/Abstract]) OR "HCC"[Title/Abstract] OR "liver cell carcinoma*"[Title/Abstract] OR "liver cancer*"[Title/Abstract] OR "carcinoma liver cell"[Title/Abstract] OR "liver carcinoma*"[Title/Abstract]) AND ("chemoembolization, therapeutic"[MeSH Terms] OR "transarterial chemoembolization"[Title/Abstract] OR "TACE"[Title/Abstract] OR "transarterial"[Title/Abstract] OR "transarterially"[Title/Abstract] OR "chemoembolic"[Title/Abstract] OR "chemoembolisation"[Title/Abstract] OR "chemoembolisations"[Title/Abstract] OR "chemoembolism"[Title/Abstract] OR "chemoembolization"[Title/Abstract] OR "chemoembolizations"[Title/Abstract] OR "chemoembolized"[Title/Abstract]) Filters: Clinical Trial, Comparative Study, Observational Study, Randomized Controlled Trial, Humans, English, from 2010 - 2023<br>(("carcinoma, hepatocellular"[MeSH Terms] OR ("carcinoma"[Title/Abstract] AND "hepatocellular"[Title/Abstract]) OR "hepatocellular carcinoma"[Title/Abstract] OR ("hepatocellular"[Title/Abstract] AND "carcinoma"[Title/Abstract]) OR "HCC"[Title/Abstract] OR "liver cell carcinoma*"[Title/Abstract] OR "liver cancer*"[Title/Abstract] OR "carcinoma liver cell"[Title/Abstract] OR "liver carcinoma*"[Title/Abstract]) AND ("chemoembolization, therapeutic"[MeSH Terms] OR "transarterial chemoembolization"[Title/Abstract] OR "TACE"[Title/Abstract] OR "transarterial"[Title/Abstract] OR "transarterially"[Title/Abstract] OR "chemoembolic"[Title/Abstract] OR "chemoembolisation"[Title/Abstract] OR "chemoembolisations"[Title/Abstract] OR "chemoembolism"[Title/Abstract] OR "chemoembolization"[Title/Abstract] OR "chemoembolizations"[Title/Abstract] OR "chemoembolized"[Title/Abstract])) AND ((clinicaltrial[Filter] OR

	comparativestudy[Filter] OR observationalstudy[Filter] OR randomizedcontrolledtrial[Filter]) AND (humans[Filter]) AND (english[Filter]) AND (2010:2021[pdat]))

Table S2: Embase database search strategy and results

**Database**	Embase
**Access date**	01-Dec-2023
**Filters applied**	Humans<br>English<br>Date: 01/01/2010-30/11/2023
**Search strategies**	Without applying filters<br>transarterial AND ('chemoembolization' OR 'chemoembolization'/exp OR chemoembolization) AND ('tace'/exp OR tace) AND ('liver cell carcinoma'/exp OR 'liver cell carcinoma') AND ('liver tumor'/exp OR 'liver tumor') AND hepatocellular AND ('carcinoma' OR 'carcinoma'/exp OR carcinoma) AND hcc<br><br>With filter<br>transarterial AND ('chemoembolization' OR 'chemoembolization'/exp OR chemoembolization) AND ('tace' OR 'tace'/exp OR tace) AND ('liver cell carcinoma'/exp OR 'liver cell carcinoma') AND ('liver tumor'/exp OR 'liver tumor') AND hepatocellular AND ('carcinoma' OR 'carcinoma'/exp OR carcinoma) AND hcc:ab,ti,kw AND [humans]/lim AND [english]/lim AND [2010-2023]/py

Table S3: Cochrane database search strategy and results

**Database**	Cochrane
**Access date**	01-Dec-2023
**Filters applied**	Years: 2010-2023<br>Type of study: trials
**Search strategies**	ID Search<br>#1 MeSH descriptor: [Carcinoma, Hepatocellular] explode all trees<br>#2 (hepatocellular NEXT carcinoma*):ti,ab,kw OR (Liver NEXT (Cancer* or tumor*)):ti,ab,kw OR (liver-cell NEXT carcinoma*):ti,ab,kw OR (Hepatocellular NEXT Carcinoma*):ti,ab,kw (Word variations have been searched)<br>#3 #1 OR #2<br>#4 (Transarterial NEXT chemoemboli*):ti,ab,kw OR (TACE):ti,ab,kw (Word variations have been searched)<br>#5 #3 AND #4

Table S4: Google scholar database search strategy and results

**Database**	Google scholar
**Access date**	01-Dec-2023
**Filters applied**	Date: 2010-2023<br>Article title search

**Search strategies**	allintitle: "transarterial chemoembolization" "hepatocellular carcinoma" "transarterial chemoembolization" "liver carcinoma" OR "hepatic cancer" OR HCC OR TACE "hepatocellular carcinoma"

Table S5: Web of Science database search strategy and results

**Database**	Web of Science
**Access date**	01-Dec-2023
**Filters applied**	topic search 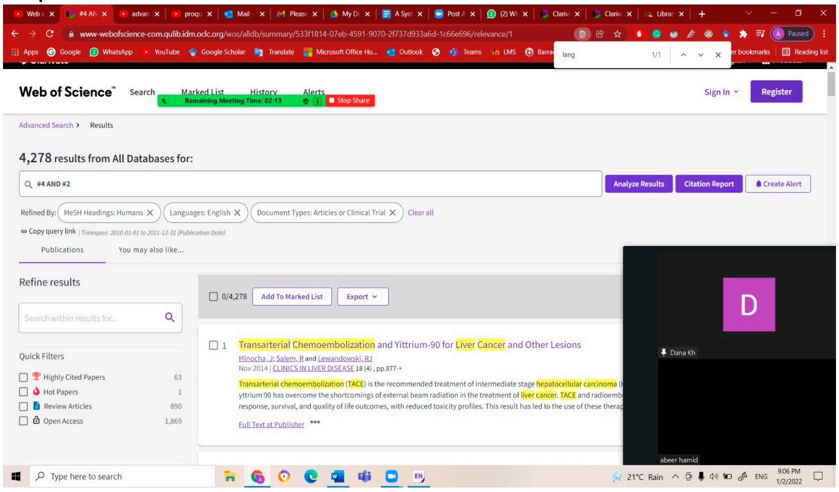

**Search strategies**	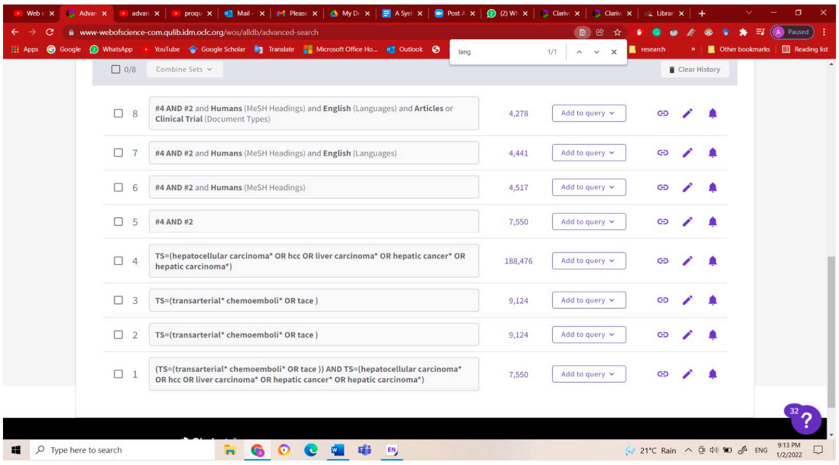 https://www.webofscience.com/wos/alldb/summary/2c27f718-5204-416d-a134-acb6971cc9a7-1fd39208/relevance/1

Gray literature

Table S6: Proquest database search strategy and results

**Database**	Proquest
**Access date**	01-Dec-2023
**Filters applied**	Date: From 01 January 2010 to 31 December 2023<br>Language: English
**Search strategies**	(su(chemoembolization, therapeutic) OR ab(transarterial chemoembolization) OR ab(TACE) OR ab(transarterial chemoemboli*)) AND (su(carcinoma hepatocellular) OR ab(hepatocellular carcinoma) OR ab(HCC) OR ab(liver cell carcinoma*) OR ab(liver carcinoma*))

Table S7: clinicaltrials.gov search strategy and results

**Database**	clinicaltrials.gov

**Access date**	01-Dec-2023
**Filters applied**	no filters
**Search strategies**	Carcinoma, Hepatocellular | hepatocellular carcinoma OR HCC OR liver cell carcinoma OR liver carcinoma | transarterial chemoembolization OR TACE OR transarterial chemoembolisation OR transarterial chemoembolic

Table S8: General details on the included studies

#	Author & Year	Aim of study	Study design	Start date	End date	Venous thrombosis
1	Huang 2022	To compare TACE-sorafenib- I-125 brachytherapy versus TACE-sorafenib in HCC patients with PVTT.	Retrospective cohort study	Jan. 2015	Dec. 2018	PVTT
2	Gao 2022	To evaluate the safety and efficacy of combined 125I seed brachytherapy and TACE for subcapsular HCC.	Retrospective cohort study	Jan. 2017	Dec. 2020	PVTT (only 4 cases in intervention and 3 cases in control)
3	Chen 2020	To assess if adding I-125 to TACE enhances efficacy in HCC patients.	Retrospective cohort study	Jan. 2014	Jul. 2017	Not mentioned
4	Luo 2016	To assess the safety and efficacy of combining EVBT with I-125 , stent placement, and TACE for treating HCC with PVTT.	Retrospective cohort study	Jan. 2009	Jan. 2014	PVTT
5	Peng 2014	To assess the efficacy and safety of lobaplatin-TACE combined with radioactive I-125 seed implantation in treating primary HCC.	Non- RCT	Jan. 2010	Feb. 2012	PVTT (Only 6 cases in intervention and 3 cases in control)
6	Wang 2021	To compare the safety and efficacy of integrated I-125 seed implantation combined with TACE versus TACE alone for HCC with PVTT.	Retrospective cohort study	Dec. 2016	Jan. 2020	PVTT
7	Sun 2018	This study evaluated the efficacy and safety of combining I-125 seed implantation with TACE for HCC patients with PVTT.	Retrospective cohort study	Jan. 2013	Jun. 2015	PVTT
8	Li 2018	To compare the therapeutic effects of I-125 combined with TACE versus TACE alone for unresectable HCC with obstructive jaundice.	Retrospective cohort study	May. 2009	Jul. 2016	Not mentioned
9	Chuan-Xing 2011	To analyze the safety and efficacy of TACE combined with portal vein stent and 125I implantation for treating PVTT in HCC.	Retrospective cohort study	Jan. 2008	Dec. 2010	PVTT
10	Yang 2014	This trial examines TACE combined with I-125 seed implantation versus conventional TACE for HCC with PVTT.	RCT	May 2011	Feb. 2013	PVTT
11	Hu 2017	To evaluate the safety and efficacy of TACE combined with CT-guided I-125 implantation in HCC patients with PVTT.	Retrospective cohort study	Jan. 2009	Dec. 2011	PVTT
12	Zhang 2018	To assess the safety and efficacy of I-125 seed implantation with TACE for hepatitis	Retrospective cohort study	January 2013	Jun. 2016	PVTT

		B-related unresectable HCC with portal vein invasion.				
13	Hong 2021	To assess the safety and efficacy of ultrasound-guided I-125 brachytherapy for HCC with PVTT.	Retrospective cohort study	2015	2019	PVTT
14	Yang 2016	This study assessed the safety and efficacy of combining TACE with irradiation stent implantation for treating HCC with IVCTT.	Retrospective cohort study	Oct. 2010	Dec. 2014	IVCTT
15	Zhang 2017	This study evaluated the combined use of EVBT, TACE, and sorafenib for treating HCC with PVTT.	Retrospective cohort study	Jan. 2009	Dec. 2015	PVTT
16	Huang 2016	This study investigated the survival benefit of TACE with I-125 implantation in hepatitis B-related HCC with PVTT.	Retrospective cohort study	Jan. 2011	Jun. 2014	PVTT
17	Li 2016	This study investigated whether combining I-125 implantation with TACE could improve overall survival in patients with HCC.	Retrospective cohort study	Jan. 2010	Dec. 2012	Not mentioned
18	Lin 2023	To evaluate the efficacy and safety of a combination therapy involving TACE, lenvatinib, a PD-1 inhibitor, and I-125 brachytherapy, compared to the control group.	Retrospective cohort study	Jul. 2017	Aug.2022	PVTT

Table 9: Summary of Meta-Analysis Results (TACE + I-125 vs. TACE Monotherapy)

Outcome Category	Specific Endpoint	N (Studies)	Estimated OR (95% CI)	Statistical Significance (p-value)	Key Findings & Heterogeneity/Bias Notes
**PRIMARY OUTCOMES: Overall Survival (OS)**	**OS at 1 Year**	k=18	3.64 (2.92–4.55)	< 0.001	The odds of survival were 3.64 times higher in the combination group. Average result significantly deviated from zero (z = 11.42). No significant heterogeneity (I^2 = 4.91%). No outliers or publication bias detected.
	OS at 1 Year (Retrospective studies)	K=16	3.66 (2.87–4.66)	< 0.001	Significant difference observed
	OS at 1 Year (Prospective studies)	K= 2	3.78 (1.34–10.67)	0.012	Significant difference observed
	OS at 1 Year (High Dose Subgroup: ≥ 100 Gy)	k=7	2.76 (1.92–3.97)	< 0.0001	Significant difference observed
	OS at 1 Year (Low Dose Subgroup: < 80 Gy)	k=3	8.55 (4.32–16.92)	< 0.0001	Significant difference observed, but lower than the high-dose group.

	OS at 1 Year (studies with systemic therapy)	k=15	3.68 (2.89–4.70)	< 0.001	Significant difference observed
	OS at 1 Year (studies without systemic therapy)	k=3	4.13 (1.69–10.09)	0.002	Significant difference observed
	**OS at 2 Years**	k=13	3.93 (2.29–6.77)	< 0.0001	Average outcome differed significantly from zero (z = 4.9432). Significant heterogeneity detected (I^2 = 62.98%, p = 0.0012). One potential outlier (Zhang 2018) identified. No publication bias.
	**OS at 3 Years**	k=10	4.12 (2.24–7.56).	< 0.001	Odds of outcome about 4.12 times higher in the exposed group. Significant heterogeneity detected (I^2 = 71.8000%, p = 0.002). One potential outlier (Zhang 2018) identified. No publication bias.
**SECONDARY OUTCOMES: Tumor Response (ORR)**	Nonspecific ORR	k=3	5.71 (1.92–16.96)	0.002	Odds of outcome 5.71 times higher in the exposed group. Moderate heterogeneity (I^2 = 54.3552%). Publication bias suggested by the regression test (p = 0.0404).
	Intrahepatic Tumor ORR	k=7	1.87 (1.33–2.63)	0.0003	Odds of outcome 1.87 times higher in the exposed group. No significant heterogeneity detected

					(I^2 = 22.4842%). No outliers or publication bias.
	PVTT/IVCTT ORR	k=11	5.63 (2.81–11.29)	< 0.0001	Odds of outcome 5.63 times higher in the exposed group. Significant heterogeneity indicated (I^2 = 73.9969%). No outliers or publication bias.
**SECONDARY OUTCOMES: Disease Control (DCR)**	Nonspecific DCR	k=2	2.83 (1.07–7.45)	0.035	Odds of outcome 2.83 times higher in the exposed group. No heterogeneity (I^2 = 0.0000%). No outliers or publication bias.
	Intrahepatic Tumor DCR	k=6	1.24 (0.54–2.83)	0.612	Not statistically significant. High heterogeneity detected (I^2 = 79.9367%, p = 0.0009). One study (Zhang et al., 2018) identified as a potential/overly influential outlier. No publication bias.
	PVTT/IVCTT DCR	k=9	4.03 (2.12–7.68)	< 0.001	Odds of outcome 4.03 times higher in the exposed group. Significant heterogeneity detected (I^2 = 73.9507%, p = 0.0005). No outliers or publication bias.
**SECONDARY OUTCOMES: Progression-Free Survival (PFS)**			Pooled Median PFS (Months)		Descriptive analysis performed as meta-analysis was not feasible. Combination therapy consistently

					resulted in longer PFS across all categories.
	Nonspecific PFS	3 (studies reported)	Gao et al. (11 vs 5 months); Chen et al. (16 vs 8 months); Luo et al. (2.4 vs 1.3 months)		Better PFS with the combination treatment compared to the control
	Intrahepatic Tumor PFS	1 (study reported)	5.0 (Combination) vs. 2.0 (TACE alone)		Data from Hong et al..
	PVTT PFS	3 (studies reported)	9.97 (Combination) vs. 5.77 (TACE alone)		Chuan-Xing et al. (7.9 vs 5.3 months); Hong et al. (9 vs 3 months); Lin et al. (13 vs 9 months)
**ADVERSE EVENTS**	Specific Event	N (Studies)	Estimated OR (Effect Size)	Statistical Significance (p-value)	Odds Ratio Interpretation (TACE + I-125 vs. TACE)
	Nausea/Vomiting	14	0.88	0.6431	Odds are 12% lower in exposed group. Considerable heterogeneity (I^2 = 75.73%).
	Diarrhea	3	0.99	0.9688	Minimal decrease in odds. No significant heterogeneity.
	Fever	14	1.08	0.5295	Slight increase in odds. No significant heterogeneity.

	Liver Abnormalities	5	0.95	0.8792	Slight decrease in odds. No statistically significant heterogeneity.
	Myelosuppression	2	1.82	0.1953	Odds are 1.82 times higher in the exposed group, but not statistically significant.
	Abdominal Pain	10	1.20	0.2171	Modest increase in odds.
	GIT Bleeding	4	1.12	0.7514	Modest increase in odds. No significant heterogeneity.
	Biloma	2	1.57	0.4661	57% increase in odds. No significant heterogeneity.
	Liver Abscesses	4	1.59	0.5509	59% increase in odds. No heterogeneity.
	Hypertension	2	0.87	0.6592	13% decrease in odds. No heterogeneity.
	Ascites	2	0.84	0.6035	16% decrease in odds. No significant heterogeneity.
	**General AE Summary**	N/A	N/A	N/A	None of the adverse events studied were substantial, and no significant side effects were reported. Publication bias was absent across all outcomes.

Table S10: Comparison of tumor response between the intervention group (TACE plus I-125) versus the control group (TACE monotherapy) in patients with hepatocellular carcinoma

Tumor Response

1) Complete Response

	Not specified				Intrahepatic Tumor				PVTT			
**Study**	**Control**	**N=75**	**Intervention**	**N=64**	**Control**	**N=502**	**Intervention**	**N=351**	**Control**	**N=577**	**Intervention**	**N=432**
Gao, 2022	6		18		NA		NA		NA		NA	
Peng, 2014	7		13		NA		NA		NA		NA	
Huang, 2022	NA		NA		0		5		0		7	
Peng, 2014	NA		NA		0		0		0		0	
Wang, 2021	NA		NA		0		6		1		7	
Chuan-Xing, 2011	NA		NA		NA		NA		0		0	
Yang, 2014	NA		NA		0		2		0		1	
Hu, 2017	NA		NA		0		0		0		0	
Zhang, 2018	NA		NA		0		0		0		0	
Hong, 2021	NA		NA		0		0		0		5	
Huang, 2016	NA		NA		0		1		0		0	
Lin, 2023	NA		NA		NA		NA		2		4	
**Total, n (%)**	**13 (17.33%)**		**31 (48.44%)**		**0 (0.00%)**		**14 (3.99%)**		**3 (0.52%)**		**24 (5.56%)**	

2) Partial Response

	Not specified				Intrahepatic Tumor				PVTT			
**Study**	**Control**	**N=75**	**Intervention**	**N=64**	**Control**	**N=502**	**Intervention**	**N=351**	**Control**	**N=577**	**Intervention**	**N=432**
Gao, 2022	13		11		NA		NA		NA		NA	
Peng, 2014	13		9		NA		NA		NA		NA	
Huang, 2022	NA		NA		30		39		11		36	
Peng, 2014	NA		NA		5		6		1		11	
Wang, 2021	NA		NA		45		45		15		20	

Chuan-Xing, 2011	NA	NA	NA	NA	8	10
Yang, 2014	NA	NA	8	8	0	8
Hu, 2017	NA	NA	36	34	1	20
Zhang, 2018	NA	NA	4	6	0	12
Hong, 2021	NA	NA	2	6	2	15
Huang, 2016	NA	NA	20	16	30	38
Lin, 2023	NA	NA	NA	NA	17	21
**Total, n (%)**	**26 (34.67%)**	**20 (31.25%)**	**150 (29.88%)**	**160 (45.58%)**	**85 (14.73%)**	**191 (44.21%)**

3) Stable Disease

	Not specified		Intrahepatic Tumor		PVTT	
**Study**	**Control N=75**	**Intervention N=64**	**Control N=460**	**Intervention N=308**	**Control N=535**	**Intervention N=389**
Gao, 2022	10	1	NA	NA	NA	NA
Peng, 2014	6	5	NA	NA	NA	NA
Huang, 2022	NA	NA	29	14	52	19
Peng, 2014	NA	NA	2	2	7	7
Wang, 2021	NA	NA	15	11	20	16
Chuan-Xing, 2011	NA	NA	NA	NA	9	13
Hu, 2017	NA	NA	8	9	8	19
Zhang, 2018	NA	NA	12	6	19	6
Hong, 2021	NA	NA	15	19	11	17
Huang, 2016	NA	NA	25	1	39	0
Lin, 2023	NA	NA	NA	NA	12	17
**Total, n (%)**	**16 (21.33%)**	**6 (9.38%)**	**106 (23.04%)**	**62 (20.13%)**	**177 (33.08%)**	**114 (29.31%)**

4) Partial Disease

	Not specified		Intrahepatic Tumor		PVTT	
**Study**	**Control N=75**	**Intervention N=64**	**Control N=322**	**Intervention N=249**	**Control N=367**	**Intervention N=304**
Gao, 2022	3	2	NA	NA	NA	NA
Peng, 2014	17	5	NA	NA	NA	NA

Huang, 2022	NA	NA	38	16	34	12
Sun, 2018	NA	NA	10	2	34	17
Hu, 2017	NA	NA	6	7	41	11
Zhang, 2018	NA	NA	40	8	37	2
Hong, 2021	NA	NA	18	9	16	3
Lin, 2023	NA	NA	NA	NA	14	13
**Total, n (%)**	**20 (26.67%)**	**7 (10.94%)**	**112 (34.78%)**	**42 (16.87%)**	**176 (47.96%)**	**58 (19.08%)**

5) Overall Response Rate

	Not specified		Intrahepatic Tumor		PVTT/IVCTT	
**Study**	**Control N=123**	**Intervention N=99**	**Control N=395**	**Intervention N=308**	**Control N=566**	**Intervention N=475**
Gao, 2022	59.4	90.7	NA	NA	NA	NA
Chen, 2020	50	68.6	NA	NA	NA	NA
Huang, 2022	NA	NA	30.9	59.5	11	58.1
Peng, 2014	NA	NA	20	28.6	4	52.4
Wang, 2021	NA	NA	0.642	0.797	0.229	0.421
Chuan-Xing, 2011	NA	NA	NA	NA	56.7	88.5
Yang, 2014	NA	NA	19.5	23.3	0	20.9
Hong, 2021	NA	NA	5.7	17.6	5.7	58.9
Yang, 2016	NA	NA	NA	NA	21.4	24.2
Lin, 2023	NA	NA	NA	NA	42.22	45.45
Peng, 2014	46.51	68.75	NA	NA	NA	NA
Hu, 2017	NA	NA	72	68	2	40
Zhang, 2017	NA	NA	NA	NA	16.1	45.9
Huang, 2016	NA	NA	14.29	24.29	21.43	54.29
**Total, (%)**	**51.97**	**79.65**	**23.29**	**31.73**	**16.43**	**44.46**

6) Disease Control Rate (%)

	Not specified		Intrahepatic Tumor		PVTT	
**Study**	**Control N=5**	**Intervention N=64**	**Control N=423**	**Intervention N=292**	**Control N=498**	**Intervention N=373**

Peng, 2014	60.47	84.37	NA	NA	NA	NA
Wang, 2021	NA	NA	28	38.1	32	85.7
Hu, 2017	NA	NA	88	86	18	78
Zhang, 2018	NA	NA	90	60	33.9	28.6
Hong, 2021	NA	NA	48.6	73.5	54.3	91.2
Zhang, 2017	NA	NA	NA	NA	29	67.6
Huang, 2016	NA	NA	32	47.1	49	76
Lin, 2023	NA	NA	NA	NA	68.88	76.37
Gao, 2022	90.63	93.75	NA	NA	NA	NA
Huang, 2022	NA	NA	60.82	78.38	64.95	83.78
Chuan-Xing, 2011	NA	NA	NA	NA	56.67	88.46
**Total, (%)**	**75.55**	**89.06**	**57.9**	**63.85**	**45.19**	**75.08**

Table Abbreviations: progression-free survival rate [PFSR], response rates (complete response rate [CRR], overall response rate [ORR], partial response rate [PRR], and disease control rate [DCR].

Table S11: PFS outcome (median months)

Study	Not specified		Intrahepatic Tumor		PVTT	
	**Control**	**Intervention**	**Control**	**Intervention**	**Control**	**Intervention**
Gao, 2022	5	11	NA	NA	NA	NA
Chen, 2020	8	16	NA	NA	NA	NA
Luo, 2016	1.3	2.4	NA	NA	NA	NA
Chuan-Xing, 2011	NA	NA	NA	NA	5.3	7.9
Hong, 2021	NA	NA	2	5	3	9
Lin, 2023	NA	NA	NA	NA	9	13
**Total, months**	**6.50**	**13.50**	**2.00**	**5.00**	**5.77**	**9.97**

Table S12: Summary of Grading of Recommendations Assessment, Development and Evaluation (GRADE) Findings

Outcome	№ of Participants (Studies)	Certainty Assessment (GRADE)	Effect Estimate OR (95% CI)	Certainty
**1-year overall survival**	1872 (18 studies)	**No serious limitations in study design[1]**; no serious inconsistency[2]; no serious indirectness[3]; no serious imprecision[4]; publication bias undetected[5]	3.64 (2.92–4.55)	**⊕⊕◯◯ LOW**
**2-year overall survival**	1300 (13 studies)	**Serious limitations in study design[1]** (downgraded one level); **serious inconsistency[6]** (downgraded one level); no serious indirectness[3]; no serious	3.93 (2.29–6.77)	**⊕◯◯◯ VERY LOW**

		imprecision[4]; publication bias undetected[7]		
**3-year overall survival**	1073 (10 studies)	**Serious limitations in study design[1]** (downgraded one level); **serious inconsistency[8]** (downgraded one level); no serious indirectness[3]; no serious imprecision[4]; publication bias undetected[7]	4.12 (2.24–7.56)	⊕◯◯◯ **VERY LOW**
**ORR (PVTT/IVCTT)**	842 (11 studies)	**Serious limitations in study design[1]** (downgraded one level); **serious inconsistency[9]** (downgraded one level); no serious indirectness[3]; no serious imprecision[4+]; publication bias undetected[10]	5.63 (2.81–11.29)	⊕◯◯◯ **VERY LOW**
**Adverse events (any)**	Range: 71–1472 (2–18 studies)	**Serious limitations in study design[1]** (downgraded one level); **serious inconsistency[11]** (downgraded one level); no serious indirectness[3]; **serious imprecision[12]** (downgraded one level)	See forest plots (Figures S7–S17)	⊕◯◯◯ **VERY LOW**

Footnotes

[1] **Limitations in study design:** Evidence predominantly from retrospective cohort studies (16 of 18) with moderate risk of bias on ROBINS-I assessment. For 1-year OS, these limitations were not considered serious enough to warrant downgrading from the starting LOW certainty, given the consistency of findings ($I^2 = 4.9\%$), large sample size (n = 1872), and absence of other concerns. For all other outcomes, the limitations were judged serious, warranting downgrading one level.

[2] **Inconsistency (1-year OS):** I^2 = 4.9%, p = 0.397; no important heterogeneity detected.

[3] **Indirectness:** All studies originated from China, which may limit applicability to non-Asian populations with different HCC etiologies (e.g., lower HBV prevalence, higher prevalence of NAFLD/alcoholic liver disease) and practice patterns. However, biological plausibility suggests findings are likely transferable; therefore not downgraded.

[4] **Imprecision (survival outcomes):** 95% confidence intervals exclude the null (OR = 1.0) and do not cross clinically important thresholds for harm; optimal information size achieved (>1000 participants for each survival outcome).

[4+] **Imprecision (ORR):** 95% confidence interval excludes the null; the lower bound (2.81) remains above the threshold for a clinically important effect (OR > 2.0). Optimal information size is borderline (842 participants) but not downgraded given the precision of the estimate.

[5] **Publication bias (1-year OS):** Funnel plot symmetrical; Begg's test p = 0.330, Egger's test p = 0.402.

[6] **Inconsistency (2-year OS):** I^2 = 63.0%, p = 0.0012; substantial unexplained heterogeneity warranting downgrading one level.

[7] **Publication bias (2- and 3-year OS):** Funnel plot assessment limited by number of studies (k = 13, k = 10); statistical tests non-significant but cannot definitively exclude bias.

[8] **Inconsistency (3-year OS):** I^2 = 71.8%, p = 0.0002; substantial unexplained heterogeneity warranting downgrading one level.

[9] **Inconsistency (ORR PVTT/IVCTT):** I^2 = 74.0%, p = 0.0001; substantial unexplained heterogeneity warranting downgrading one level.

[10] **Publication bias (ORR PVTT/IVCTT):** Funnel plot symmetrical; Begg's test p = 0.218, Egger's test p = 0.051.

[11] **Inconsistency (adverse events):** Significant heterogeneity was observed for several events (e.g., nausea/vomiting I^2 = 75.7%, p < 0.001; fever I^2 = 0%, p = 0.892). Downgraded one level for serious inconsistency across the body of evidence.

[12] **Imprecision (adverse events):** Confidence intervals for most adverse events included the null (OR = 1.0) and crossed clinically important thresholds for both benefit and harm. Sample sizes for some events (e.g., biloma, n = 71; hypertension, n = 118) were below the optimal information size.

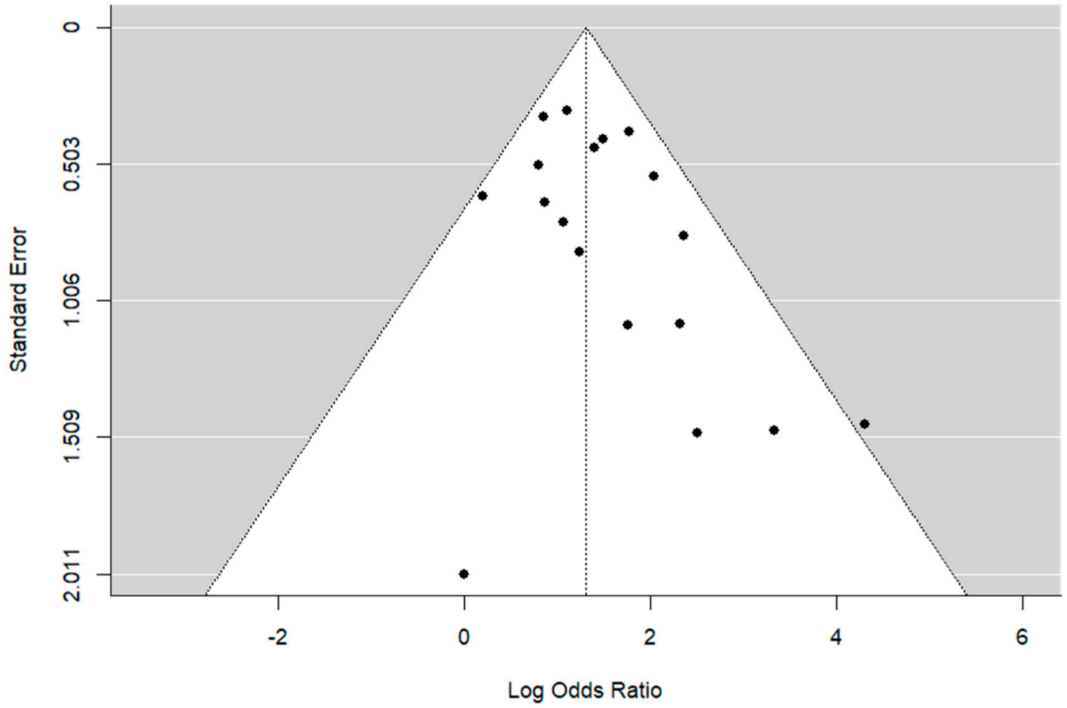

Figure S1: Regression Test for Funnel Plot Asymmetry_OS at 1 year from all included studies

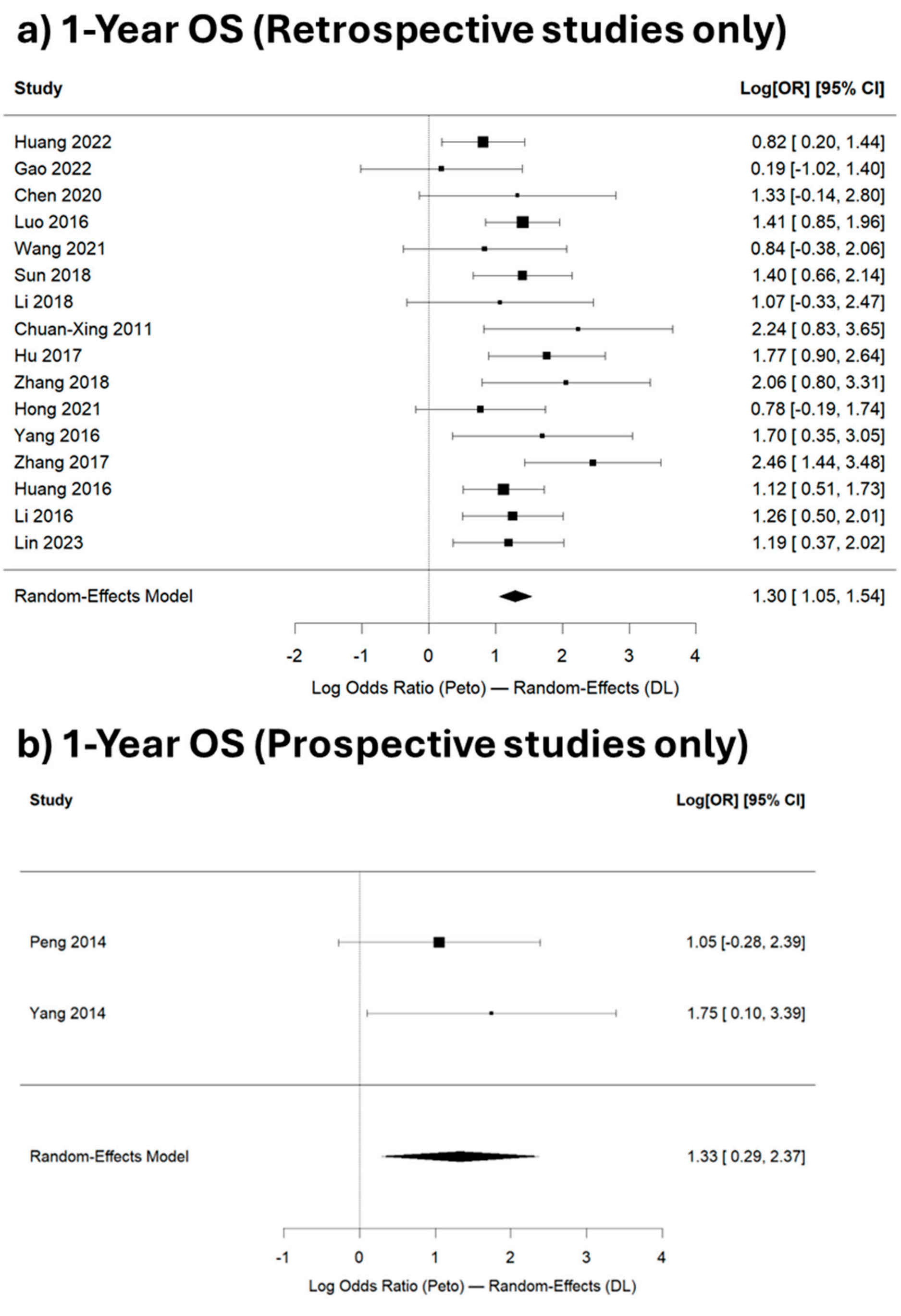

Figure S2: Comparison of the 1-year OS rates between the control and intervention groups. (a) Retrospective studies only; (b) Prospective studies only. NB: All analyses were conducted using the random-effects model.

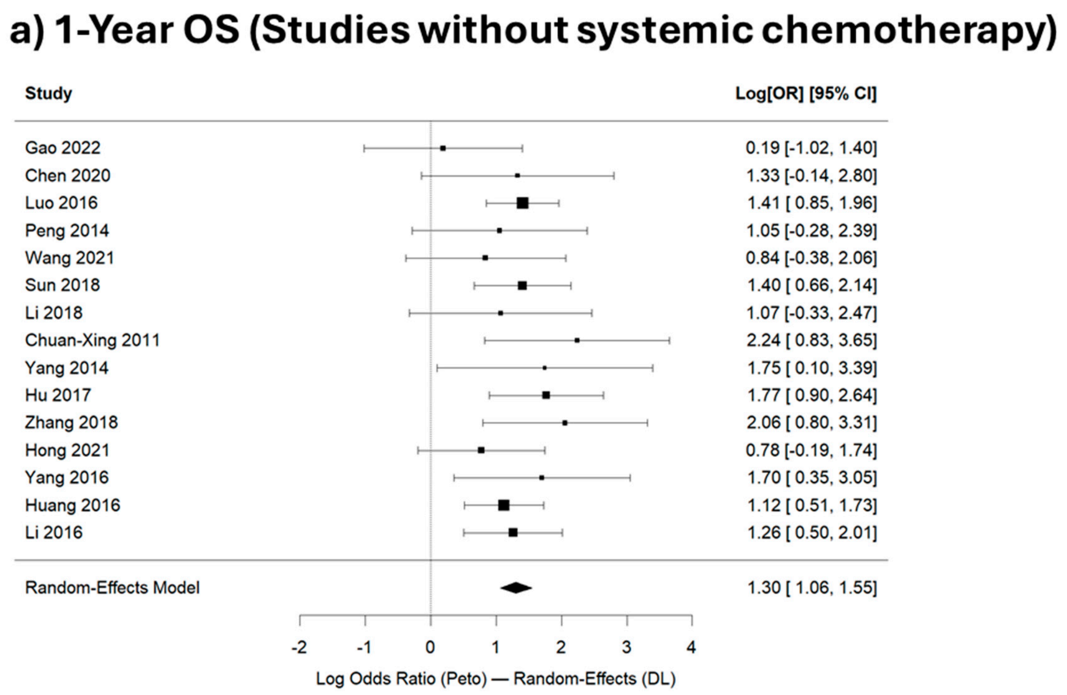

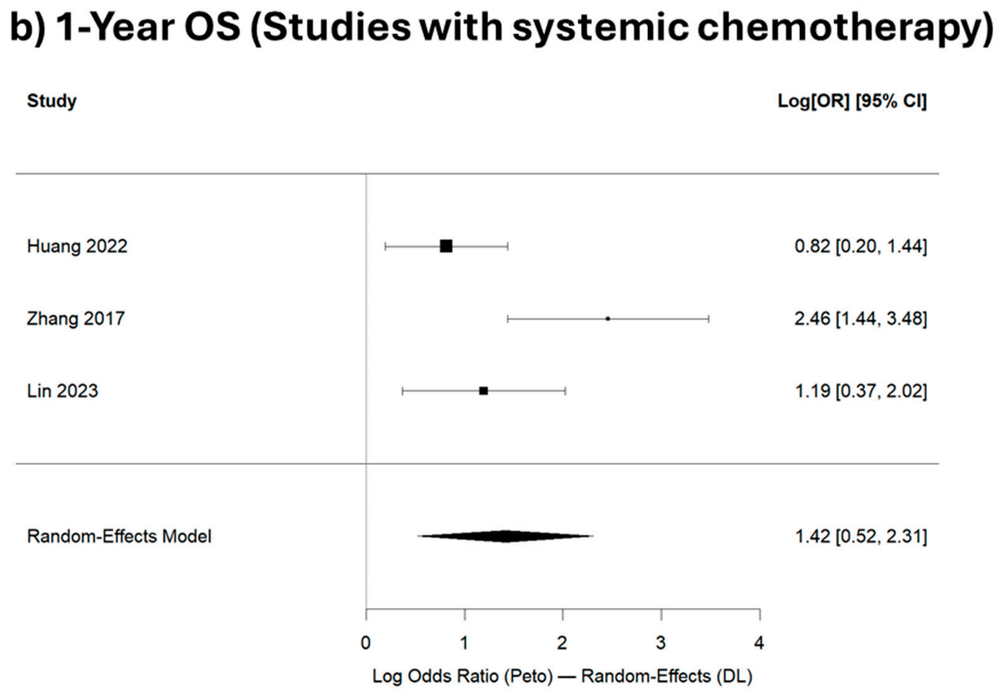

Figure S3: Comparison of the 1-year OS rates between the control and intervention groups. (a) Studies without systemic chemotherapy; b) Studies with systemic chemotherapy. NB: All analyses were conducted using the random-effects model.

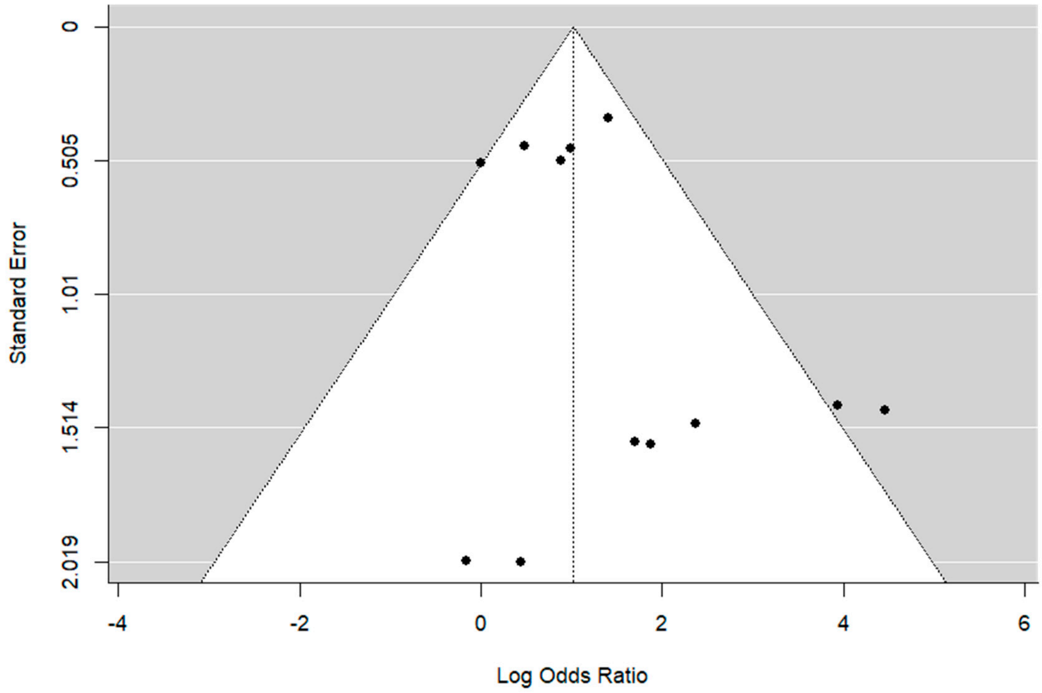

Figure S4: Regression Test for Funnel Plot Asymmetry_OS at 2 year from included studies

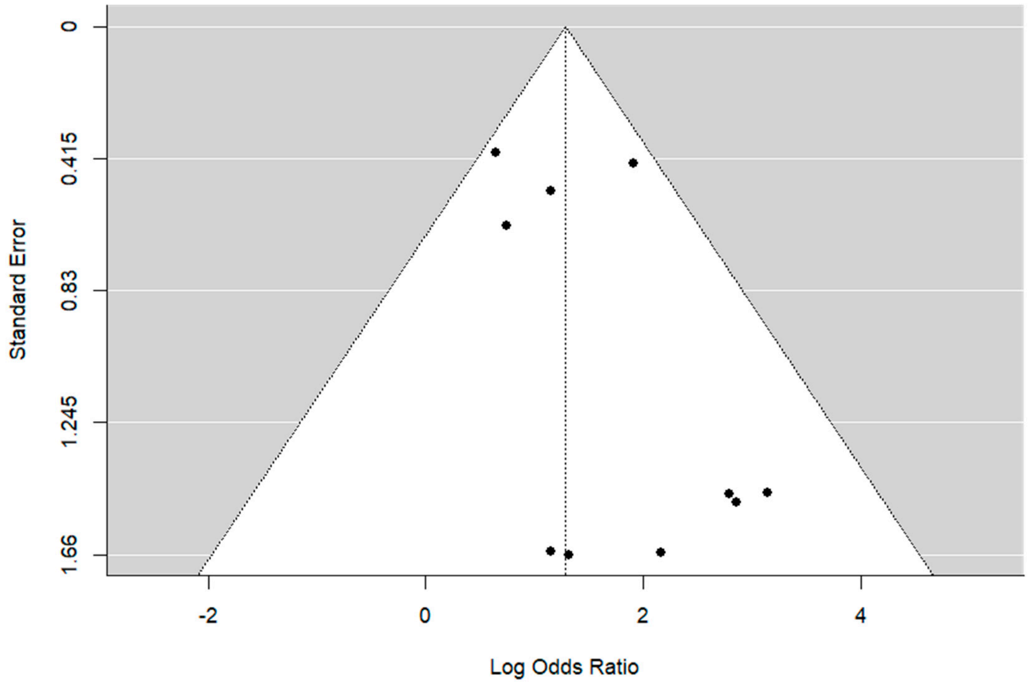

Figure S5: Regression Test for Funnel Plot Asymmetry_OS at 3 year from included studies

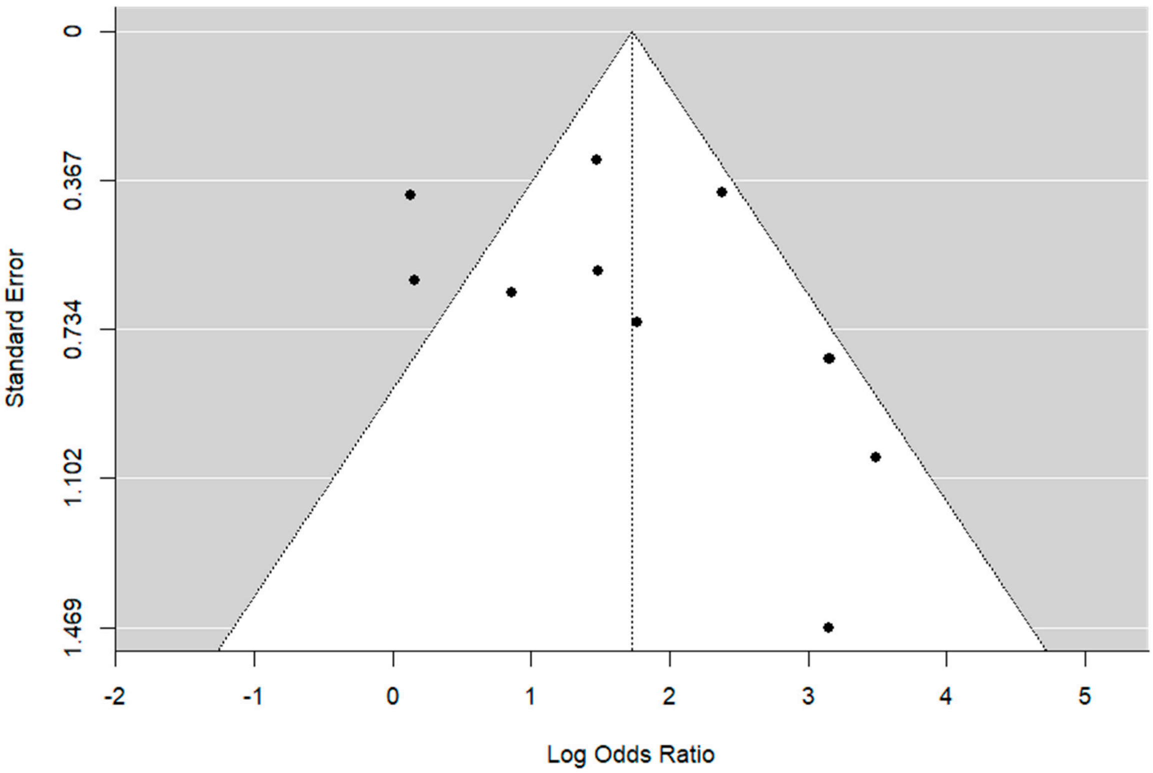

Figure S6: Regression Test for Funnel Plot Asymmetry_ *PVTT/IVCTT ORR*

A) Forest blot for Nausea/vomiting

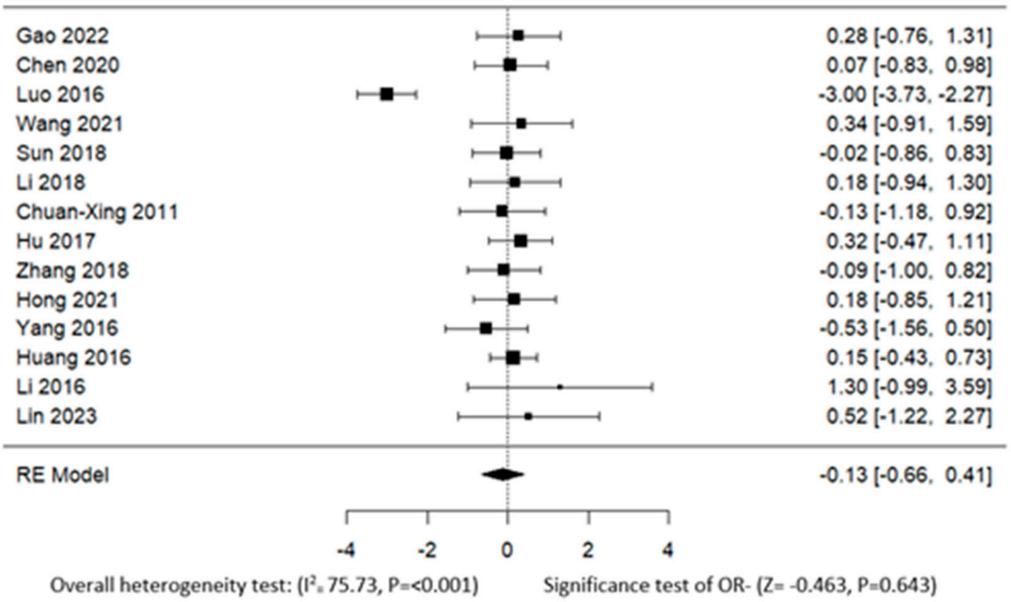

B) Funnel plot and regression test

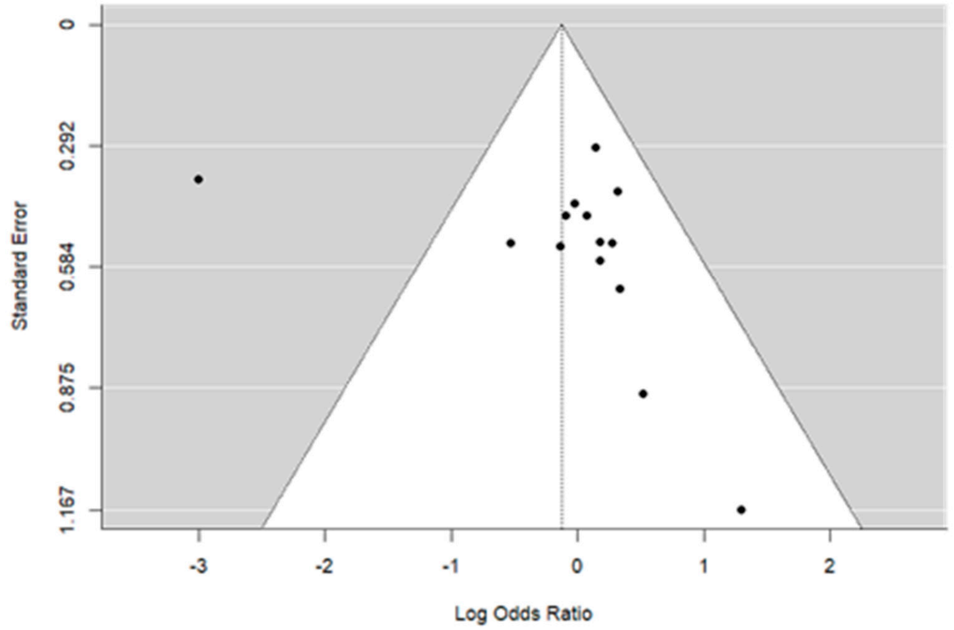

Figure S7: A) Forest plot of log odds ratios with 95% confidence intervals for the analysis of nausea and vomiting in the intervention versus control groups. **(B)** Regression test for funnel plot asymmetry among studies included in the nausea and vomiting outcome analysis.

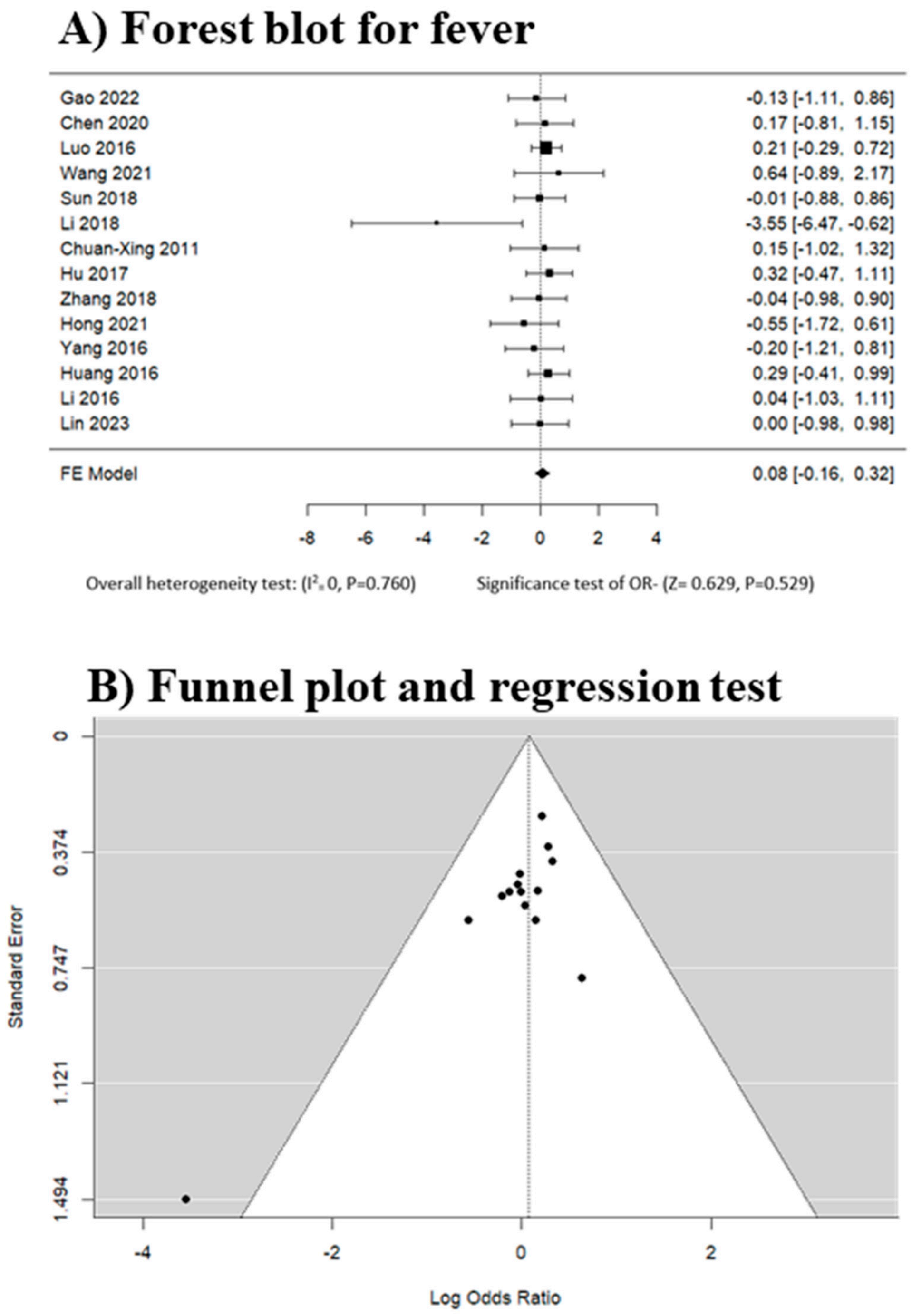

Figure S8: A) Forest plot of log odds ratios with 95% confidence intervals for the analysis of fever in the intervention versus control groups. **(B)** Regression test for funnel plot asymmetry among studies included in the fever outcome analysis.

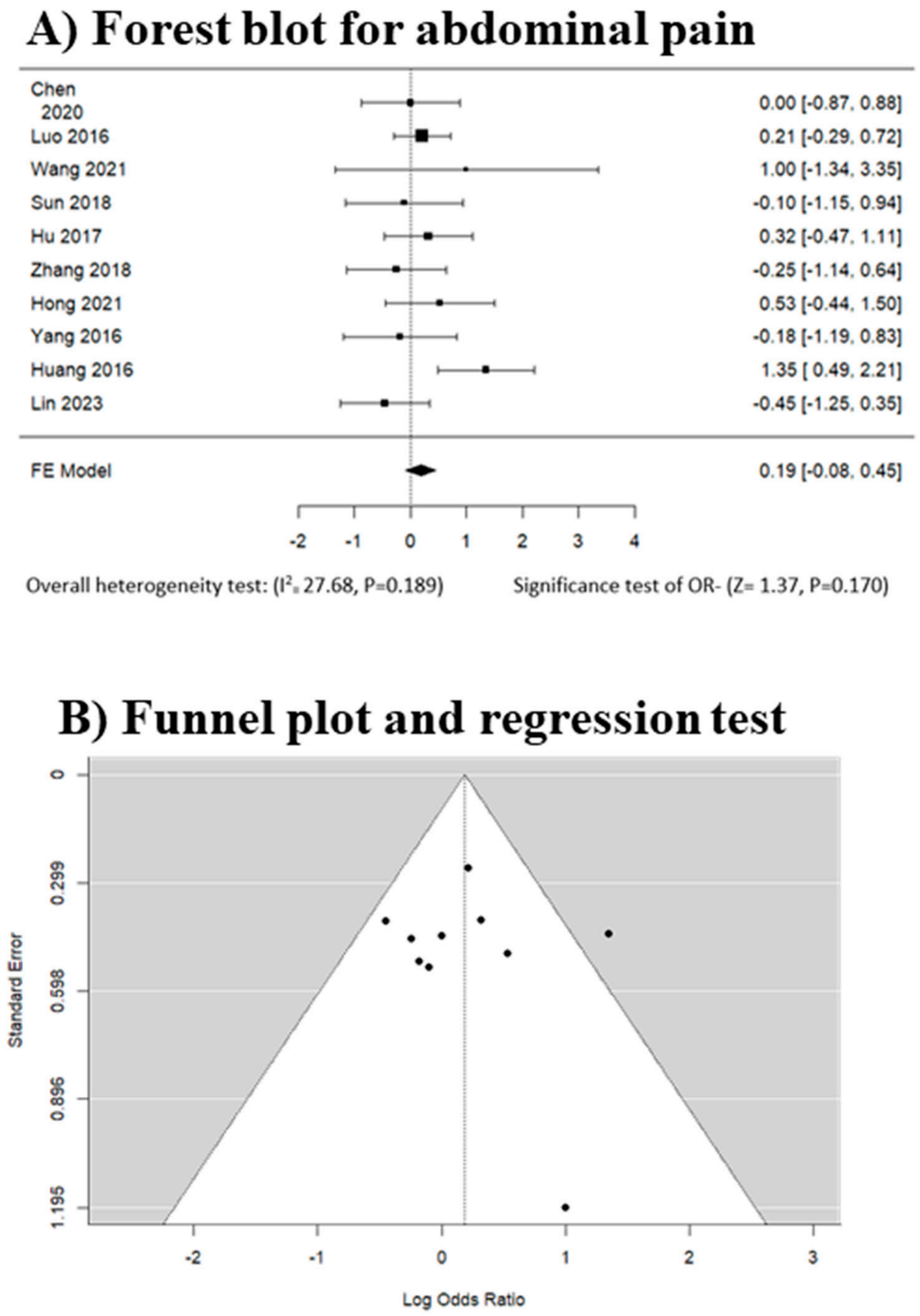

Figure S9: A) Forest plot of log odds ratios with 95% confidence intervals for the analysis of abdominal pain in the intervention versus control groups. **(B)** Regression test for funnel plot asymmetry among studies included in the abdominal pain outcome analysis.

Diarrhea

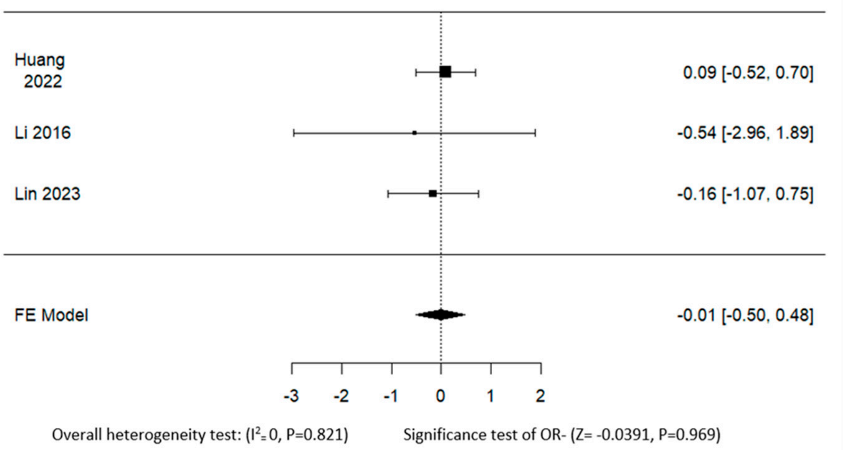

Figure S10: Forest plot of log odds ratios with 95% confidence intervals for the analysis of diarrhea in the intervention versus control groups.

Liver abnormalities

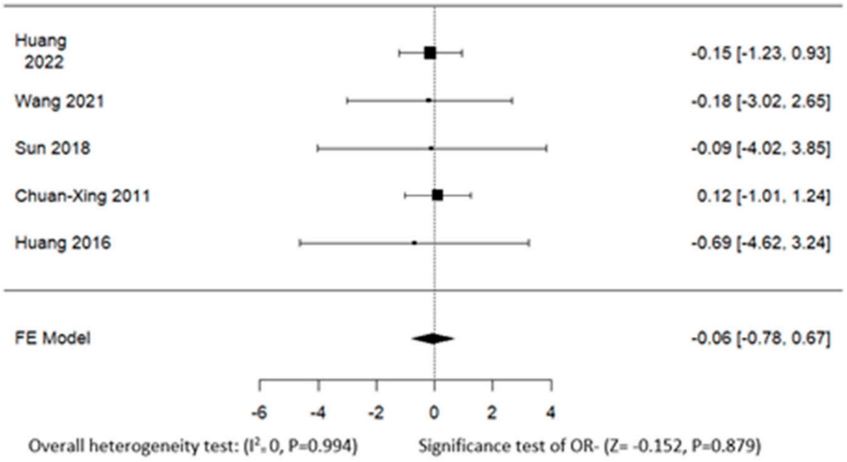

Figure S11: Forest plot of log odds ratios with 95% confidence intervals for the analysis of liver abnormalities in the intervention versus control groups.

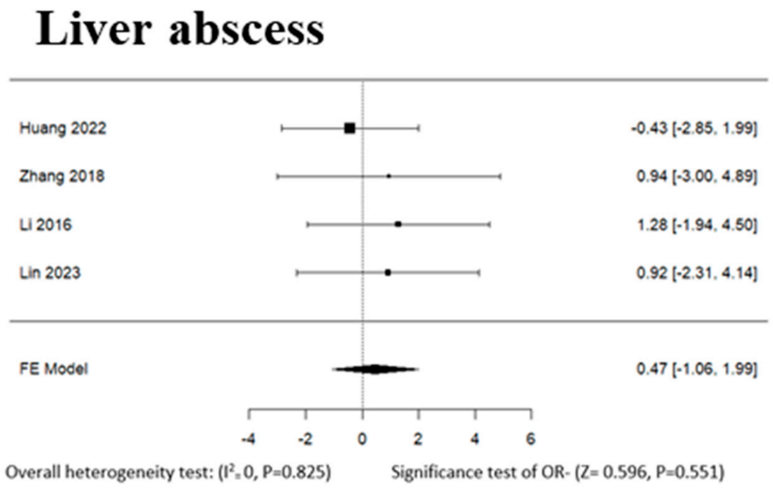

Figure S12: Forest plot of log odds ratios with 95% confidence intervals for the analysis of liver abscess in the intervention versus control groups.

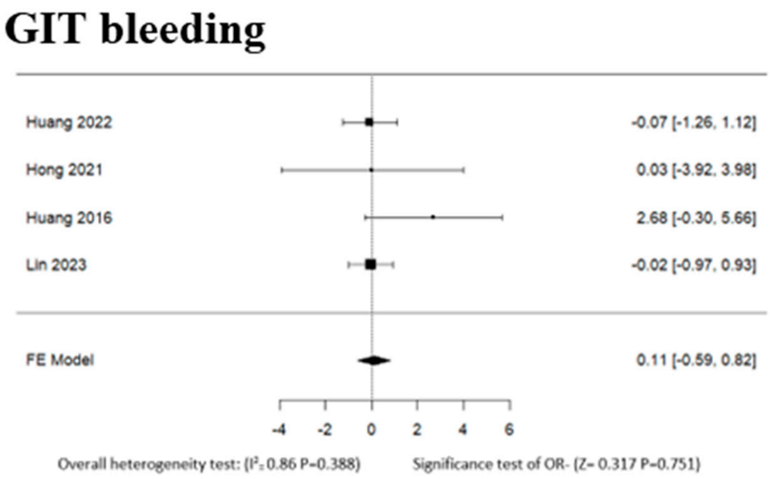

Figure S13: Forest plot of log odds ratios with 95% confidence intervals for the analysis of GIT bleeding in the intervention versus control groups.

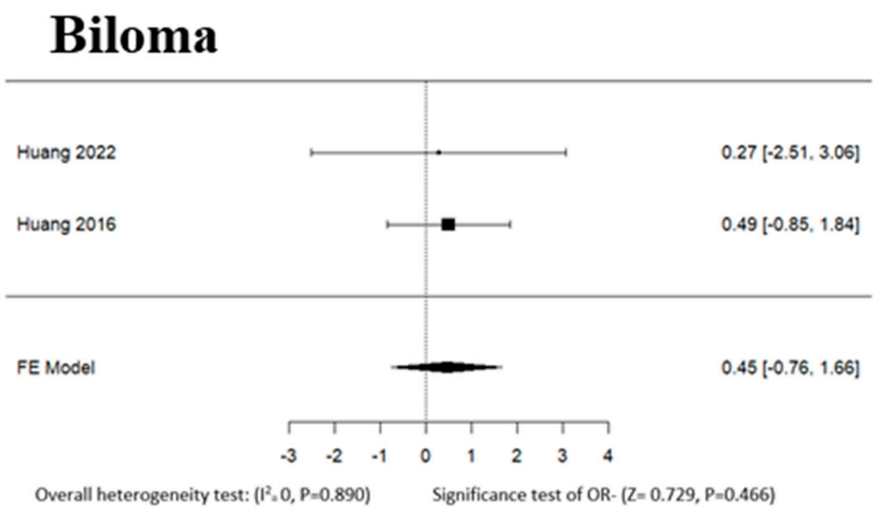

Figure S14: Forest plot of log odds ratios with 95% confidence intervals for the analysis of biloma in the intervention versus control groups.

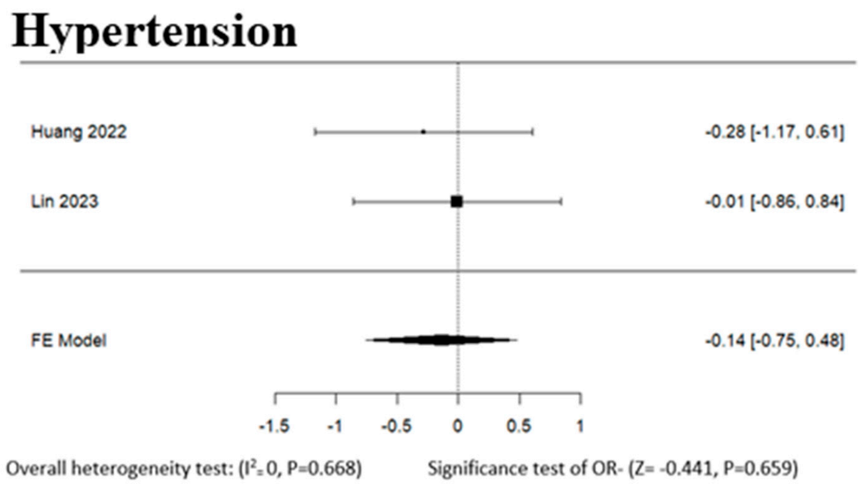

Figure S15: Forest plot of log odds ratios with 95% confidence intervals for the analysis of hypertension in the intervention versus control groups.

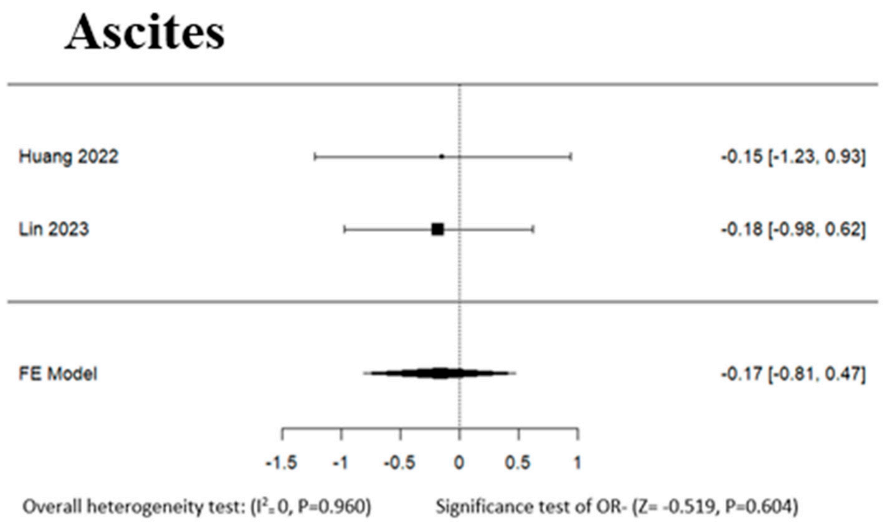

Figure S16: Forest plot of log odds ratios with 95% confidence intervals for the analysis of ascites in the intervention versus control groups.

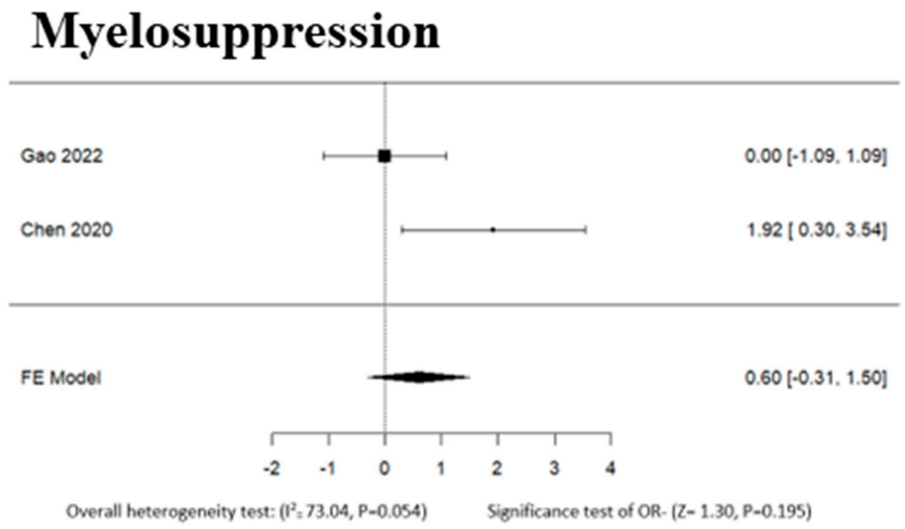

Figure S17: Forest plot of log odds ratios with 95% confidence intervals for the analysis of myelosuppression in the intervention versus control groups.
